# CSF1R^+^ Macrophages Sustain Pancreatic Tumor Growth through T Cell Suppression and Maintenance of Key Gene Programs that Define the Squamous Subtype

**DOI:** 10.1016/j.celrep.2018.03.131

**Published:** 2018-05-02

**Authors:** Juliana B. Candido, Jennifer P. Morton, Peter Bailey, Andrew D. Campbell, Saadia A. Karim, Thomas Jamieson, Laura Lapienyte, Aarthi Gopinathan, William Clark, Ewan J. McGhee, Jun Wang, Monica Escorcio-Correia, Raphael Zollinger, Rozita Roshani, Lisa Drew, Loveena Rishi, Rebecca Arkell, T.R. Jeffry Evans, Colin Nixon, Duncan I. Jodrell, Robert W. Wilkinson, Andrew V. Biankin, Simon T. Barry, Frances R. Balkwill, Owen J. Sansom

**Affiliations:** 1 Barts Cancer Institute, Queen Mary University of London, London EC1M 6BQ, UK; 2 Cancer Research UK Beatson Institute, Glasgow G61 1BD, UK; 3 Institute of Cancer Sciences, University of Glasgow, Glasgow G61 1QH, UK; 4 Cancer Research UK Cambridge Institute, Li Ka Shing Centre, University of Cambridge, Robinson Way, Cambridge CB2 0RE, UK; 5 Bioscience, Oncology, iMED Biotech Unit, AstraZeneca, Boston, MA, USA; 6 MedImmune Ltd, Granta Park, Cambridge CB21 6GH, UK; 7 Bioscience, Oncology, iMED Biotech Unit, AstraZeneca, Cambridge, UK

**Keywords:** pancreatic cancer, macrophages, CSF1R, T cells

## Abstract

Pancreatic ductal adenocarcinoma (PDAC) is resistant to most therapies including single-agent immunotherapy and has a dense desmoplastic stroma, and most patients present with advanced metastatic disease. We reveal that macrophages are the dominant leukocyte population both in human PDAC stroma and autochthonous models, with an important functional contribution to the squamous subtype of human PDAC. We targeted macrophages in a genetic PDAC model using AZD7507, a potent selective inhibitor of CSF1R. AZD7507 caused shrinkage of established tumors and increased mouse survival in this difficult-to-treat model. Malignant cell proliferation diminished, with increased cell death and an enhanced T cell immune response. Loss of macrophages rewired other features of the TME, with global changes in gene expression akin to switching PDAC subtypes. These changes were markedly different to those elicited when neutrophils were targeted via CXCR2. These results suggest targeting the myeloid cell axis may be particularly efficacious in PDAC, especially with CSF1R inhibitors.

## Highlights

• Macrophages functionally contribute to the squamous subtype of human PDAC
• Inhibition of CSF1R alters the TME and results in an enhanced T cell immune response
• Loss of macrophages rewires PDAC gene expression and switches subtype
• Macrophage inhibition induces changes markedly different to neutrophil targeting

## Graphical Abstract

**Figure:**
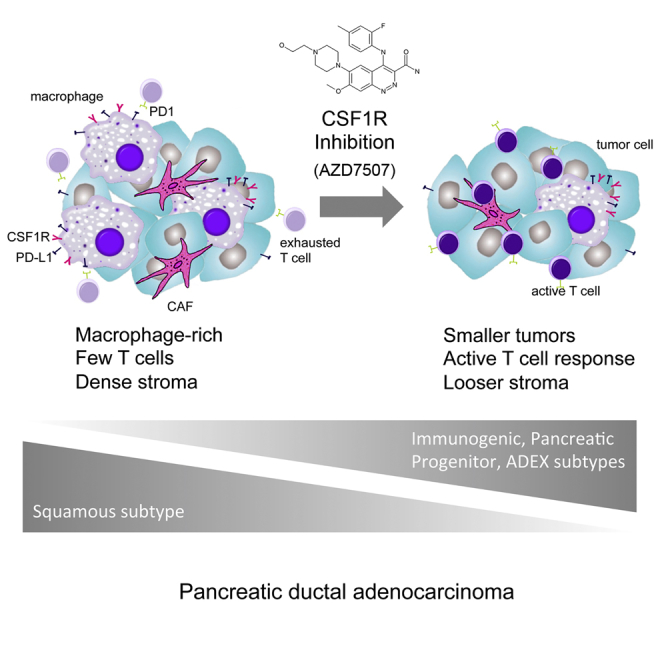

## Teaser

Candido et al. find that CSF-1R^+^ macrophages play a vital role in pancreatic tumor maintenance, suppression of T cells, and tumor gene networks based on transcriptome analysis. In an autochthonous model, CSF-1R inhibition alters the gene expression programs that influence subtype specification of PDAC and extends survival.

## Introduction

Pancreatic cancer is predicted to be the second most common cause of cancer death by 2030 (Rahib et al., 2014). The major reason for this is that, unlike many other cancers, there have been no significant improvements in survival over the past 30 years. That said, over the last 5 years there has been great progress in the understanding of pancreatic cancer (both pre-clinically and clinically) with numerous innovative trials currently under way.

Many of these new developments are based on a deep understanding of the molecular pathology of pancreatic cancer. The definition of subtypes and the use of autochthonous mouse models have allowed modeling of tumor-stroma interaction, and the metastatic process, in immune competent mice (Gopinathan et al., 2015). The most studied model is the “KPC” mouse model of PDAC when KRAS^G12D^ and P53^R172H^ are targeted to the murine pancreas (normally using the *Pdx1-Cre*) (Hingorani et al., 2005). Several studies in mice have highlighted the importance of the stroma, which provides important pro-survival cues and may impair therapeutic responsiveness (Neesse et al., 2015).

Human and murine pancreatic ductal adenocarcinoma (PDAC) is characterized by a significant stroma containing fibroblasts, leukocytes, and collagen deposits (Clark et al., 2007, Korc, 2007). Of the leukocyte populations, macrophages are the dominant subtype in the tumor microenvironment (TME) (Noy and Pollard, 2014). Macrophage infiltration correlates with poor prognosis in many cancer types (Qian and Pollard, 2010), including PDAC (Bailey et al., 2016, Ino et al., 2013, Knudsen et al., 2017). Recent studies have shown that targeting factors in the stroma, for example, hyaluronic acid (HA) (Provenzano et al., 2012), and LOX (Miller et al., 2015) as well as genetic ablation of genes such as *Stat3* (Corcoran et al., 2011) can slow murine PDAC. Early phase trials investigating some of these concepts are now planned or under way including compounds targeting HA (NCT02715804, NCT02921022, NCT02910882) and JAK1 (NCT02646748, NCT02265510). This is not to say that all stromal elements are tumor promoting, as a number of studies targeting the stroma have shown that this may accelerate tumorigenesis, or at least generate pancreatic tumors that are less dependent on stromal signals (Özdemir et al., 2014, Rhim et al., 2014).

Initial studies of immunotherapy using checkpoint inhibitors in human PDAC are not encouraging; however, preclinical studies have suggested co-targeting of additional stroma elements with combinations may improve efficacy. This has been observed in the autochthonous KPC model when fibroblasts are depleted (Feig et al., 2013). In addition, we (Steele et al., 2016) and others (Chao et al., 2016, Stromnes et al., 2014) have shown similar phenotypes when neutrophils/myeloid-derived suppressor cells (MDSC) are lost. Experiments using orthotopic transplantable PDAC models also show similar effects if macrophages are depleted or polarized to a tumoricidal phenotype (Beatty et al., 2011, Luheshi et al., 2016, Zhang et al., 2017, Zhu et al., 2014). Given that both macrophages and neutrophils are targets in auto-immune and inflammatory diseases, there are a number of clinic-ready drugs that could be rapidly progressed to clinical testing in PDAC in combination with checkpoint inhibition. However, we don’t yet know how different myeloid cell types influence tumor progression and response to therapy and thus have little information guiding the optimization of patient selection and treatment strategies required for successful translation to the clinic.

Recent genomic analysis suggests that pancreatic cancer can be divided into 4 different subtypes (Bailey et al., 2016): squamous, ADEX, progenitor, and immunogenic. A more detailed examination of the genes that contribute to these different subtypes suggests immunogenic and squamous subtypes show high expression of macrophage markers. Moreover, a macrophage gene expression program is associated with prognosis in these patients, consistent with a previous study (Knudsen et al., 2017). Interestingly, in contrast to other cancers, the “immunogenic” subtype does not confer an improved prognosis providing further evidence that immunotherapy as a single agent is unlikely to be effective in this disease. A recent study attempted to align the different subtypes of PDAC from multiple studies (TCGA Research Network, 2017). Here, the squamous subtype we identified was similar to the previously identified “quasi-mesenchymal subtype” of PDAC (Collisson et al., 2011). Some debate remains, however, over the “ADEX” or “Exocrine-like” subtype, with a recent TCGA study suggesting this might reflect “contamination” with non-neoplastic tissue (TCGA Research Network, 2017).

RNA sequencing (RNA-seq) of KPC tumors has allowed them to be compared with the different human subtypes. Interestingly, our analysis has shown that there are elements of all the subtypes within the KPC tumors. One of the hallmarks of the squamous subtype of PDAC are gene programs associated with squamous differentiation, hypoxia, extracellular matrix and transforming growth factor β (TGF-β) signaling. Importantly, we have shown that these are dependent on mutant p53, so mice that lack p53 or p63 no longer express these gene programs (unlike the KPC) and show reduced or absent metastasis. The fact that KPC mice have these multiple subtypes allows us to see whether any specific subtype is affected by therapeutic intervention.

Tumor-associated macrophages (TAMs) play an important role not only in tumor progression and metastasis but also in resistance to chemotherapy and radiotherapy (De Palma and Lewis, 2013, Qian and Pollard, 2010). Preclinical models have elucidated the critical role of TAMs in cancer development, progression, and metastasis (DeNardo et al., 2011, Mantovani et al., 2017, Nielsen et al., 2016, Xu et al., 2013). Signaling through the cellular receptor for CSF1, CSF1R, promotes the differentiation of myeloid progenitors into populations of monocytes, macrophages, dendritic cells, and osteoclasts. Several therapeutic applications to impair recruitment or to readjust their behavior are currently being evaluated (Cannarile et al., 2017, Mantovani et al., 2017).

Given this wealth of preclinical data and the importance of macrophage signatures in the human PDAC data, we wanted to address the impact of CSF1R inhibition on established tumors in the genetic KPC PDAC mouse model and differentiate these effects from CXCR2 and CCR2 inhibition. Of all interventions studied, we found that CSF1R inhibition has the most profound effect causing tumor regression and T cell activation, independent of PD1 inhibition. Importantly, loss of macrophages in the tumor microenvironment significantly altered tumor architecture both transcriptionally and histologically. Squamous gene expression programs were downregulated, with marked activation of ADEX and immunogenic gene programs. Importantly, ADEX gene expression programs were observed within the tumor cells, thus excluding contribution of non-neoplastic contamination to the gene programs that define the ADEX subtype. Comparison with CXCR2 inhibition under the same conditions showed that the active transcriptional programs reflected the divergent effects of inhibiting specific myeloid cell populations.

## Results

### Human and Murine Primary PDACs Are Infiltrated by CSF1R^+^ Macrophages and Overexpress CSF1 and IL-34

**Figure 1:**
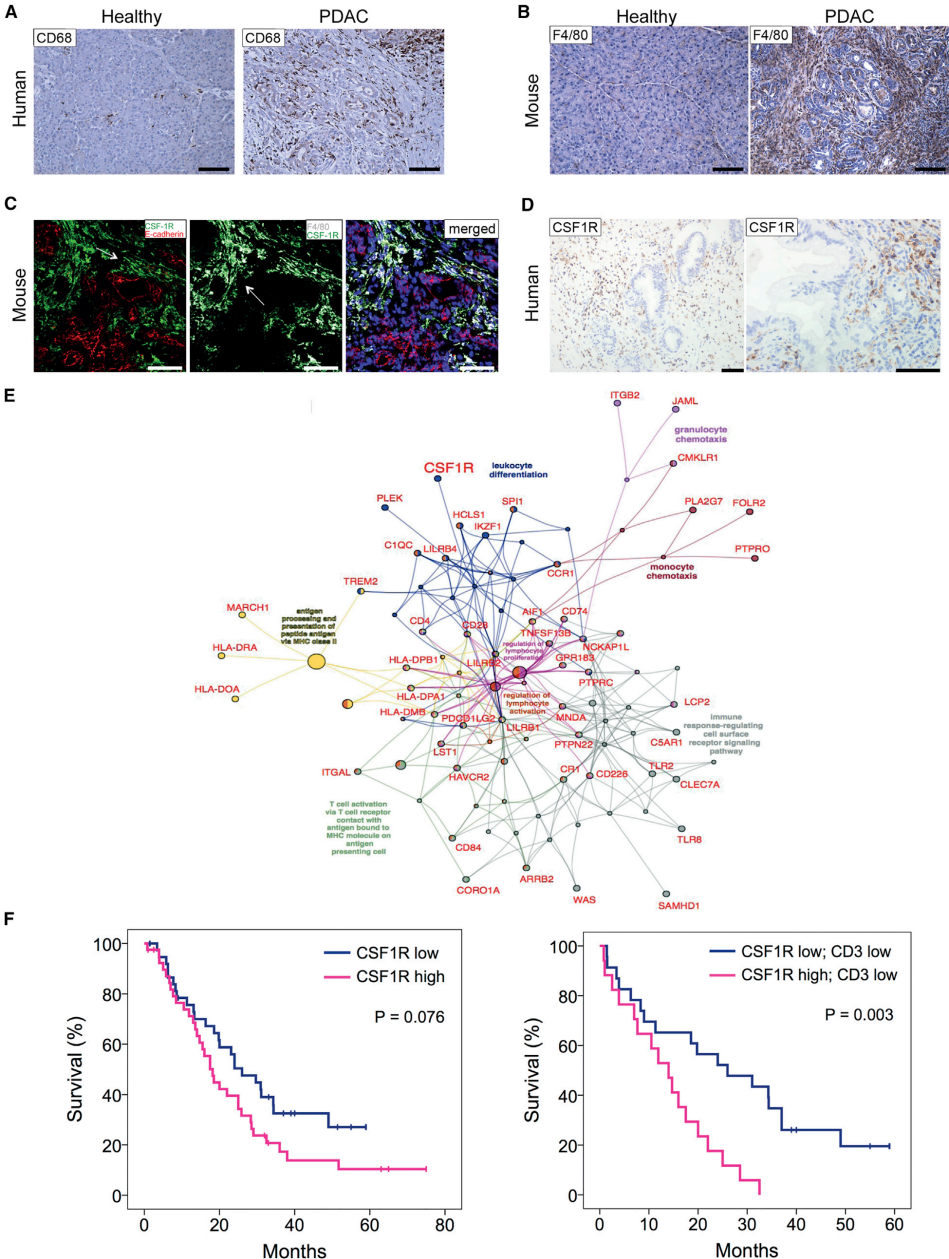
Human and Mouse PDAC Tumors Are Infiltrated by Macrophages and Overexpress CSF1R (A) IHC showing pancreas areas of positivity for CD68 in adjacent normal pancreas and patients diagnosed with PDAC. (B) IHC showing F4/80 staining in murine healthy pancreas and in PDAC tissue. Scale bar, 100 μm. (C) Immunofluorescence analysis of CSF1R in murine pancreatic tissue. Tissue sections from the pancreas of KPC mice were stained with F4/80 (gray) and E-cadherin (red), and with CSF1R (green) and counterstained with DAPI (blue). Scale bar, 100 μm. (D) IHC showing CSF1R staining in human PDAC tissue. Scale bar, 200 μm. (E) ClueGO-CluePedia functional network of the human PDAC macrophage gene program (GP7) showing genes associated with significant gene ontology terms (p value <0.01). Network nodes and edges are colored by a specific gene ontology terms. (F) Kaplan-Meier analysis of survival post-resection of a cohort of human PDAC patients in terms of low (below median) or high (above median) CSF1R expression, low or high CD3^+^ cells, and low or high CSF1R expression in CD3 low patients. p values, log rank test, n = 79. See also Figure S1.

In our previous studies, we found that the macrophage transcriptional signature was high in squamous and immunogenic subtypes of PDAC and that the macrophage transcriptional gene program was associated with a poor prognosis (Bailey et al., 2016). To determine the presence of macrophages in human PDAC tissues, we used immunohistochemistry (IHC) to stain for CD68, a transmembrane glycoprotein highly expressed by human monocytes and tissue macrophages. CD68^+^ macrophages were rarely present in normal pancreas but PDAC contained significant myeloid cell populations (Figure 1A). In the mouse, F4/80 is expressed by the majority of mature macrophages and is widely used to identify this population using immunostaining. When compared to healthy control pancreas, there was a prominent infiltration of F4/80 cells in the stroma of pancreatic tumors of KPC mice especially in macrophages accumulated around epithelial tumor cells (Figure 1B). We next measured the expression of colony-stimulating factor-1 receptor (CSF1R) on TAMs in murine pancreatic tumors. CSF1R is located predominantly on myeloid cells and is involved in macrophage recruitment. Tissues were stained with antibodies against CSF1R, F4/80 to define macrophages, and E-cadherin to identify epithelial tumor cells (Figure 1C). Co-staining of E-cadherin^+^ epithelial cells, F4/80^+^ macrophages, and CSF1R confirmed that the receptor is exclusively expressed on F4/80^+^ macrophages and not on malignant cells (Figure 1C). CSF1R protein also localized to the stroma in human PDAC biopsies (Figure 1D). CSF1R expression was also absent in PDAC tumor cell lines derived from mouse (Figure S1A) and human (Figure S1B). Tissue samples from KPC mice (Figure S1C) and patients (Figure S1D) with PDAC were further stained with immunofluorescence antibodies to evaluate the presence of CSF1R ligands, CSF1, and interleukin-34 (IL-34) in the tumor microenvironment. Patient tissues were stained with antibodies against CD68 for macrophages, pan-cytokeratin for epithelial tumor cells, and with either CSF1 or IL-34. Similar to the mouse model (Figure S1C), IL-34 and CSF1 were present in the human PDAC microenvironment (Figure S1D). Co-localization indicated that pancreatic epithelial tumor cells produce both CSF1 and IL-34 in mouse and human. In addition, there was a significant increase of CSF1 in the plasma of PDAC patients and mice when compared to aged-matched healthy individuals (Figures S1E and S1F). To confirm that tumor cells express CSF1, we performed ELISA on 3 KPC cell lines and showed high expression (Figure S1G).

Taken together these results showed significant infiltration of CSF1R-expressing macrophages in human and mouse pancreatic tumors and that the malignant cells were the source of CSF1R ligands. Importantly, CSF1R was a component of the macrophage gene expression profile identified in human PDAC and therefore offered a way to specifically target macrophages in PDAC (Figure 1E).

Intriguingly, when we assessed CSF1R expression by IHC of 79 PDAC patients, we found that high CSF1R expression was itself associated with poor prognosis, and this was particularly significant in patients with low T cell infiltration, which was not itself prognostic (Figure 1F). This is consistent with the expression of CSF1R across different subtypes of PDAC being high in both squamous and immunogenic.

### Small-Molecule Inhibitor of CSF1R AZD7507 Blocks CSF1R Phosphorylation and Depletes Macrophages *In Vitro* and *In Vivo*

In order to gain deeper insight into the role of macrophages in pancreatic cancer, we investigated the therapeutic impact of inhibiting myeloid cell recruitment using AZD7507 (Scott et al., 2013), a small-molecule tight binding ATP-competitive inhibitor of CSF1R signaling, the structure of which is available at https://pubchem.ncbi.nlm.nih.gov/compound/25001557. AZD7507 is a selective and potent inhibitor of CSF1R kinase activity with an IC_50_ of 3 nM and negligible activity against other kinases tested in *in vitro* kinase assays (Table S1).

**Figure 2:**
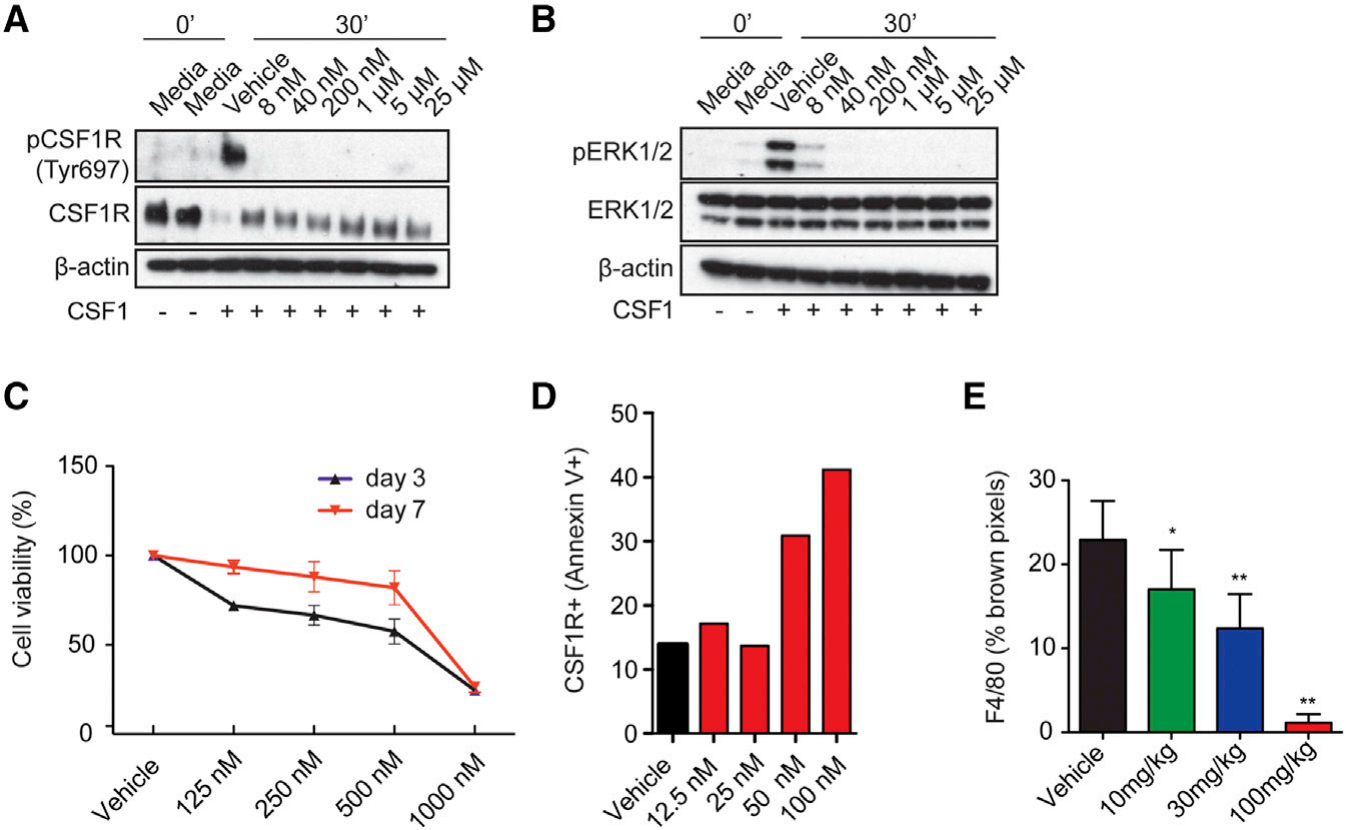
AZD7507 Is a Potent and Highly Selective ATP-Competitive Inhibitor of CSF1R Kinase Activity (A and B) Bone marrow-derived macrophages (BMDMs) were cultured with either media or CSF1 at indicated AZD7507 concentrations and protein lysate analyzed by western blot for pCSF1R (Tyr697) and (Tyr807), total CSF1R and β-actin (A), and pERK1/2 (Thr202/Tyr204), total ERK1/2 and β-actin (B). (C) Bone marrow cells were cultured with CSF1 at indicated AZD7507 concentrations for 3 and 7 days, and cell growth was assessed by the WST-1 colorimetric assay. The graph shows the percentage viability of treated cells. Data are represented as mean ± SEM for n > 3. (D) Dead CSF1R positive BMDM cells were examined using Annexin V^+^ membrane viability dye staining (FVD) and detected by flow cytometry when the cells were cultured for 7 days *in vitro* with CSF1. The graph shows the percentage of necrotic (Annexin V^+^, FVD^+^, CSF1R^+^) cells. (E) AZD7507-treated MDA-MB-231 xenograft tumors at day 20 were stained with an antibody against F4/80 for IHC, and macrophage numbers were quantified as percentage of brown pixels. Data are represented as mean ± SEM. See also Figure S2.

Western blotting confirmed that AZD7507 inhibits CSF1-induced CSF1R phosphorylation in primary bone marrow derived cells/monocytes (Figure 2A) and blocks downstream activation of ERK (Figure 2B). In addition, AZD7507 induced apoptosis of CSF1R-positive wild-type murine bone marrow cells (Figures 2C and 2D) but did not affect the viability of cells differentiated from the bone marrow in the presence of CSF1 (Figure S2A). Further, in murine PDAC cells, which did not express CSF1R (Figure S2B), AZD7507 had no effect on ERK1/2 phosphorylation (Figure S2C) or proliferation (Figure S2D).

*In vivo*, AD7507 treatment elicited a dose-dependent depletion of macrophages in MDA-MB-231 xenografts, as assessed by F4/80 IHC, with maximal depletion at 100 mg/kg (Figure 2E).

### AZD7507 Depletes Macrophages in Mice and Mediates Tumor Regression and Increases Survival

**Figure 3:**
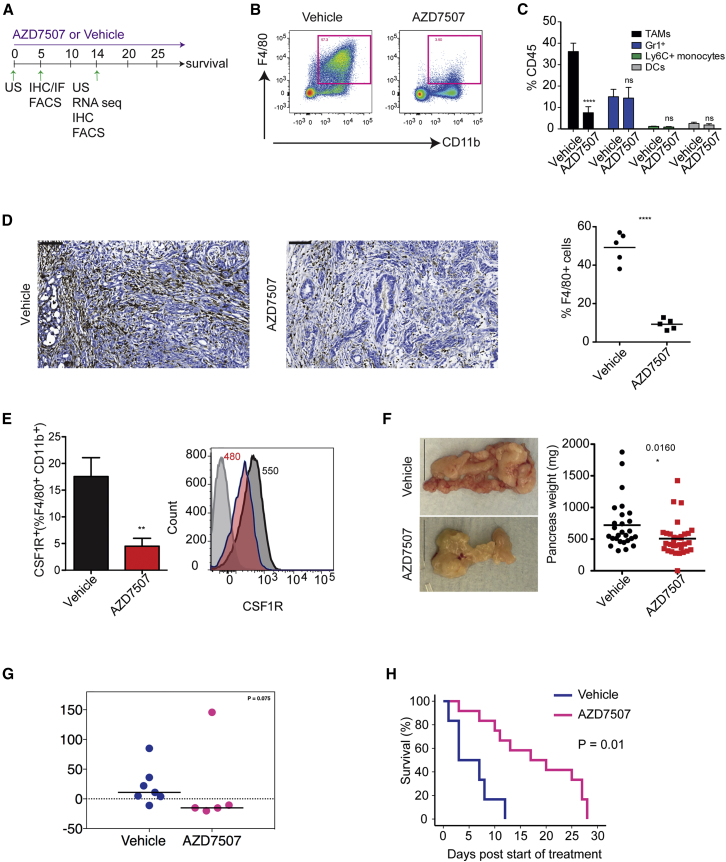
CSF1R Inhibition Depletes Macrophages, Regresses Tumor Growth, and Prolongs Survival in KPC Mice (A) Schematic diagram of the experimental setup with an outline summary of the treatment regimen and time points used. (B) Representative flow cytometry plot of CD11b and F4/80 in live CD45^+^ cells from KPC tumor of either vehicle or AZD7507-treated mice. (C) Graph shows quantification of TAMs, Gr1^+^ cells, Ly6C^+^ monocytes, and CD11c^+^ dendritic cells as percentage of total CD45^+^ cells (data are shown are mean ± SEM, n > 7, unpaired t test). (D) IHC showing F4/80^+^ macrophages in tumors from KPC mice treated as indicated. Graph shows quantification of F4/80 IHC (data are shown as mean, n = 5). (E) Histogram on right shows representative flow cytometry with numbers showing mean geoMFI data (n = 5 mice per group; isotype control, light gray; vehicle, dark gray; AZD7507, red). Graph on left shows frequency of CSF1R^+^ TAMs in tumors of mice treated as indicated (data are shown as mean ± SEM, n = 5). (F) Gross morphology of tumors and tumor weight in each group after 2 weeks of treatment. (Data shown are mean ± SEM, n > 34, unpaired t test.) (G) Tumor growth was monitored by high-resolution ultrasound in KPC mice with mean tumor diameters >5 mm before (day 0) and 14 days after treatment with either vehicle or AZD7507. Quantification of tumor volume in vehicle-treated (n = 7) and AZD7507-treated mice (n = 5). (H) Kaplan-Meier survival analysis of KPC mice treated with vehicle (n = 6) or AZD7507 (n = 12). See also Figure S3.

Having established 100 mg/kg of AZD7507 as the relevant dose to achieve macrophage depletion, we studied its activity in the KPC genetically engineered mouse model of pancreatic cancer by treating mice with established tumors with AZD7507 (scheduling is shown in Figure 3A).

Flow cytometry analysis (Figure S3A) of tumor tissue after 2 weeks of AZD7507 treatment revealed a significant reduction in the frequency of F4/80^+^CD11b^+^ TAMs (Figures 3B and 3C), with no significant effect on Ly6C^+^ monocytes, CD11c^+^ dendritic cells, or Gr1^+^ cells (Figure 3C). These results were further supported and quantified by IHC, showing that, at 2 weeks after treatment, the percentage of F4/80^+^ TAMs was significantly reduced (Figure 3D) and accompanied by a relative decrease in both CSF1R expression and CSF1R^+^ myeloid cells (Figure 3E). RNA-seq of tumors suggested that the macrophages that remained resembled M1 type, as expression signatures for M1 were relatively enriched in AZD7507-treated mice compared to vehicle (Figure S3B).

We observed that *in vivo* administration of AZD7507 caused a reduction in tumor mass after 2 weeks of treatment (Figure 3F), and this was confirmed by ultrasound (US) imaging (Figure 3G). Importantly, this was associated with an increase in overall survival (Figure 3H). It is important to note that established tumors in the KPC model are resistant to many agents e.g., gemcitabine, Mek inhibition, PI3K inhibition, and rapamycin and very few therapies work as single agents (Alagesan et al., 2015, Gopinathan et al., 2015, Morran et al., 2014, Olive et al., 2009).

### CSF1R Inhibition Alters Tumor Cell Proliferation and the Stromal Microenvironment

In line with the reduction of TAMs after AZD7507 detected by IHC, Affymetrix mRNA expression array analysis of tumors after 5 days of treatment showed downregulated expression of genes characteristic of macrophage infiltration, including *Csf1r*, *Arg1*, *Emr1*, *Mrc1*, and *Msr1* (Figure S4A). The upregulated genes were enriched for molecules associated with T cell phenotype and immune activation, including *Cd69*, *CD8*, and *Gzma*, suggesting an increase in local adaptive immunity (Figure S4B).

**Figure 4:**
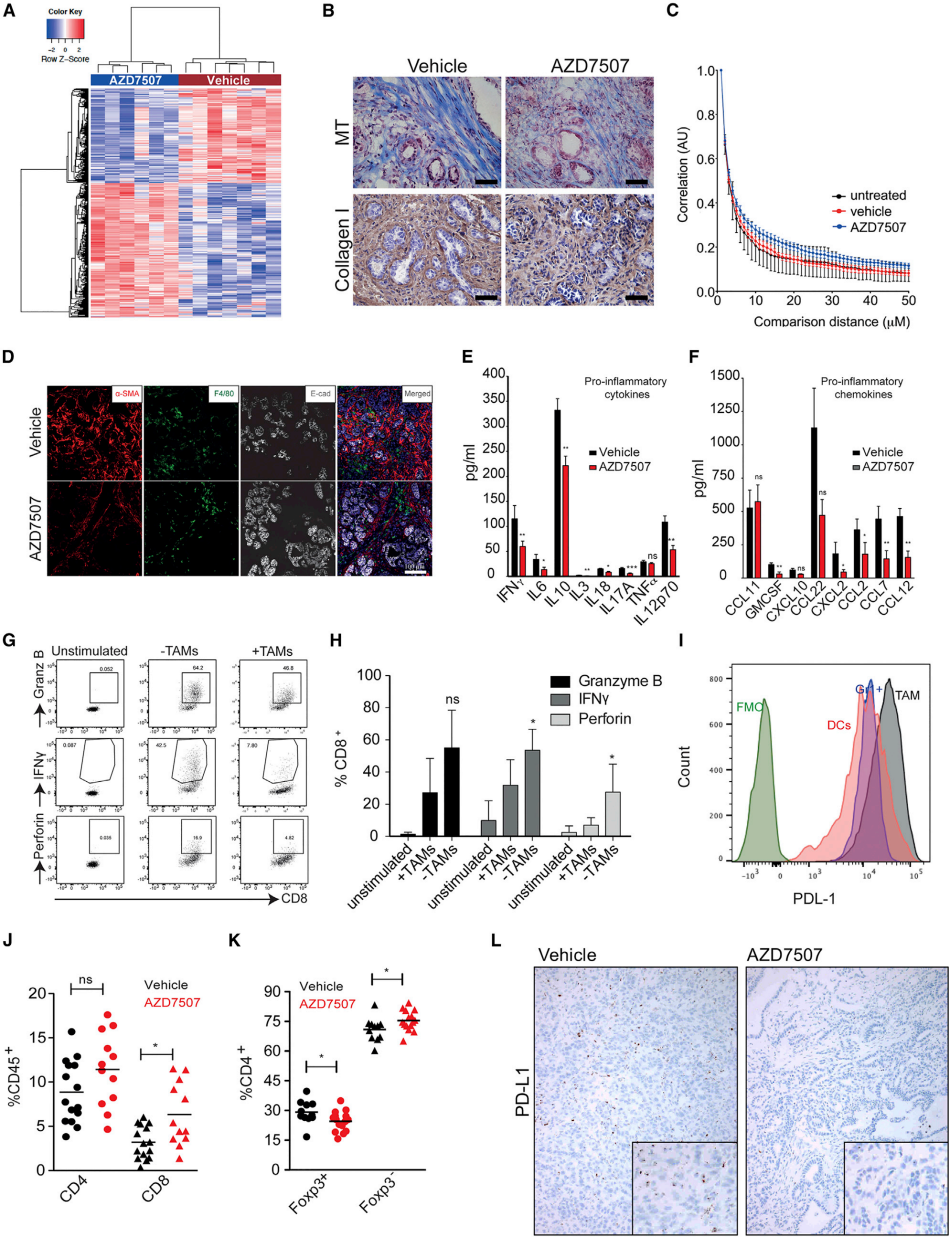
AZD7507 Alters Tumor Microenvironment Composition and Function (A) Signal log2 expression of downregulated (blue) and upregulated (red) genes in vehicle-treated tumors compared with AZD7507. The color intensity is proportional to the signal log2 intensity. All genes had a false discovery rate (FDR) <0.05. (B) IHC staining of Masson’s Trichrome and Collagen I in tumors of KPC mice treated for 5 days with either vehicle or AZD7507 (n > 3). Bar, 50 μm. (C) Gray-level correlation matrix (GLCM) texture analysis of the second harmonic generation (SHG) signal emitted by PDAC-associated collagen in mice treated as indicated. Mean ± SEM at each distance. (D) KPC tissue sections from the pancreas of each group were stained against E-cadherin (gray) and with α-SMA (red) and F4/80 (green). Tissues were counterstained with the nuclear stain DAPI (blue). Image represents tissue samples from a total of at least n = 3 KPC mice per group. (E and F) Protein lysates were obtained from tumors treated as indicated and were analyzed for cytokine contents using multiplex (E), cytokine (F), and chemokine analysis (Myriad RBM Mouse Inflammation MAO v.1.0 array) (n > 9). (G and H) Representative flow cytometry analysis (G) and quantification (H) for Granzyme B, IFN-γ, and Perforin expression in either unstimulated or stimulated (CD3/CD28) cells cultured for 72 hr in the presence or absence of TAMs (n > 4). (I) Histogram of PD-L1 expression by fluorescence-activated cell sorting (FACS) analysis on myeloid subsets as indicated (n = 4). (J) Flow cytometry analysis of the CD45^+^ T cell subsets in the tumor of mice treated as indicated (n > 10). (K) Flow cytometry analysis of the frequency of Foxp3^+^ Tregs and Foxp3^–^ Effector CD4^+^ T cells in the tumor of mice treated with either vehicle or AZD7507. p values were calculated by Mann-Whitney U test (^∗^p < 0.05, ^∗∗^p < 0.01, and ^∗∗∗^p < 0.001), mean ± SEM. (L) RNAscope in site hybridization for PD-L1 in tumors for KPC mice treated with either vehicle or AZD7507. See also Figures S1 and S4.

We also analyzed the mRNA expression profile of the tumor following 14 days of treatment with either AZD7507 or vehicle control. RNA-seq analyses of whole-tumor tissue mRNA expression revealed 374 downregulated and 453 upregulated genes in AZD7507-treated tumors compared to controls (Figure 4A). The list of downregulated genes was enriched for markers involved in cell-cycle regulation (*MKi67*, *Cdk6*, *Aurka*, and *Cdc20*), (Figure S4C) DNA damage response (*Lig1*, *Pcna*, *Pole*, and *Pttg1*) (Figure S4D), and hypoxia/metabolism (*Hif3a*, *Anxa*, *Aldoa*, and *Ldha*) (Figure S4E).

We previously reported that a hypoxia gene expression signature correlated with poor prognosis of patients with PDAC following surgery, and that an important component of that signature was the collagen cross-linking enzyme LOX (Miller et al., 2015). We therefore examined changes in the tumor microenvironment of the KPC mice following AZD7507 treatment. Using Masson’s Trichrome staining, we observed a decrease in collagen I staining consistent with degradation of the tumor matrix as early as 5 days post-treatment (Figure 4B). Moreover, analysis of second harmonic signals generated through multiphoton microscopy demonstrated that AZD7507 treatment had a qualitative impact upon collagen deposition in PDAC, with both the scale and discrete nature of collagen fibrils reduced in treated specimens (Figure 4C). Fibro-inflammatory stroma is a hallmark in pancreatic cancer and may play a role in treatment resistance (Olive et al., 2009). Next, we used immunofluorescence to measure the pancreatic fibro-inflammatory stromal content by assessing alpha-smooth muscle actin (SMA) expression, a marker of activated fibroblasts. KPC mice treated with AZD7507 displayed less prominent alpha-SMA-positive stromal expansion than in vehicle-treated KPC mice (Figure 4D).

Together, these findings infer that macrophages may favor the proliferation of tumor cells as well as maintaining their survival. In addition, the data suggest that macrophages favor higher stromal expression of collagen and this may impair adaptive immune cells from exerting their effector function, thus enhancing tumor growth.

It is well established that the cytokine milieu generates an inflammatory and immunosuppressive microenvironment by inducing macrophages and Tregs to facilitate tumor progression (Mantovani et al., 2017). To assess the soluble factors in the tumor microenvironment, tumors were harvested and protein lysates were analyzed for cytokine changes. There were significant decreases in several potent pro-tumor cytokines including IL-6 and IL-10 (Figure 4E) and chemokines such as CCL2 and CCL12 (Figure 4F) in the tumors of the KPC mice treated with AZD7507 for 14 days.

To evaluate whether the high number of macrophages present in the tumor microenvironment of the KPC mice contribute to the production of these cytokines as well as their capacity to suppress T cells, we removed F4/80^+^ macrophages from tumor cell suspensions obtained from collagenase-digested tumors and then cultured the cells from the tumor in the presence of plate-bound anti-CD3 and soluble CD28. After 72 hr, cells were stained and analyzed by flow cytometry. We observed that the frequency of interferon (IFN)-γ, Granzyme B, and Perforin CD3/CD28-activated CD8^+^ T cells were increased in the absence of macrophages when compared to samples where macrophages were present (Figures 4G and 4H). This suggests that macrophages have a direct effect on the ability of T cells to become cytotoxic. In line with this hypothesis, we observed that PD-L1 was highly expressed on TAMs (Figure 4I).

As shown here, pancreatic tumor macrophages have a significant immunosuppressive capacity. To determine whether macrophage ablation would trigger anti-tumor T cell activity in PDAC tumors, we investigated tumor-infiltrating lymphocytes in KPC mice treated with AZD7507. Flow cytometry analysis of tumor-infiltrating T cells in mice treated with AZD7507 showed significantly increased CD3^+^ T cells in KPC tumors (Figure S4F). This increase correlated with significant increases in the percentages of CD3^+^CD4^+^ T cells and CD3^+^CD8 cytotoxic T cells (Figure 4J) but, importantly, no increase of CD4^+^Foxp3 T regulatory cells (Treg) (Figure 4K). Indeed, AZD7507 treatment significantly improved effector (CD4^+^Foxp3^–^)-to-Treg ratios (Figure S4G). Importantly following AZD7507 treatment, all PD-L1^+^ stroma cells were depleted suggesting the macrophages as the sole source of PD-L1 in the stroma (Figure 4L).

### CSF1R Inhibition of Macrophage Population Is Distinct from Inhibition of Other Myeloid Populations

**Figure 5:**
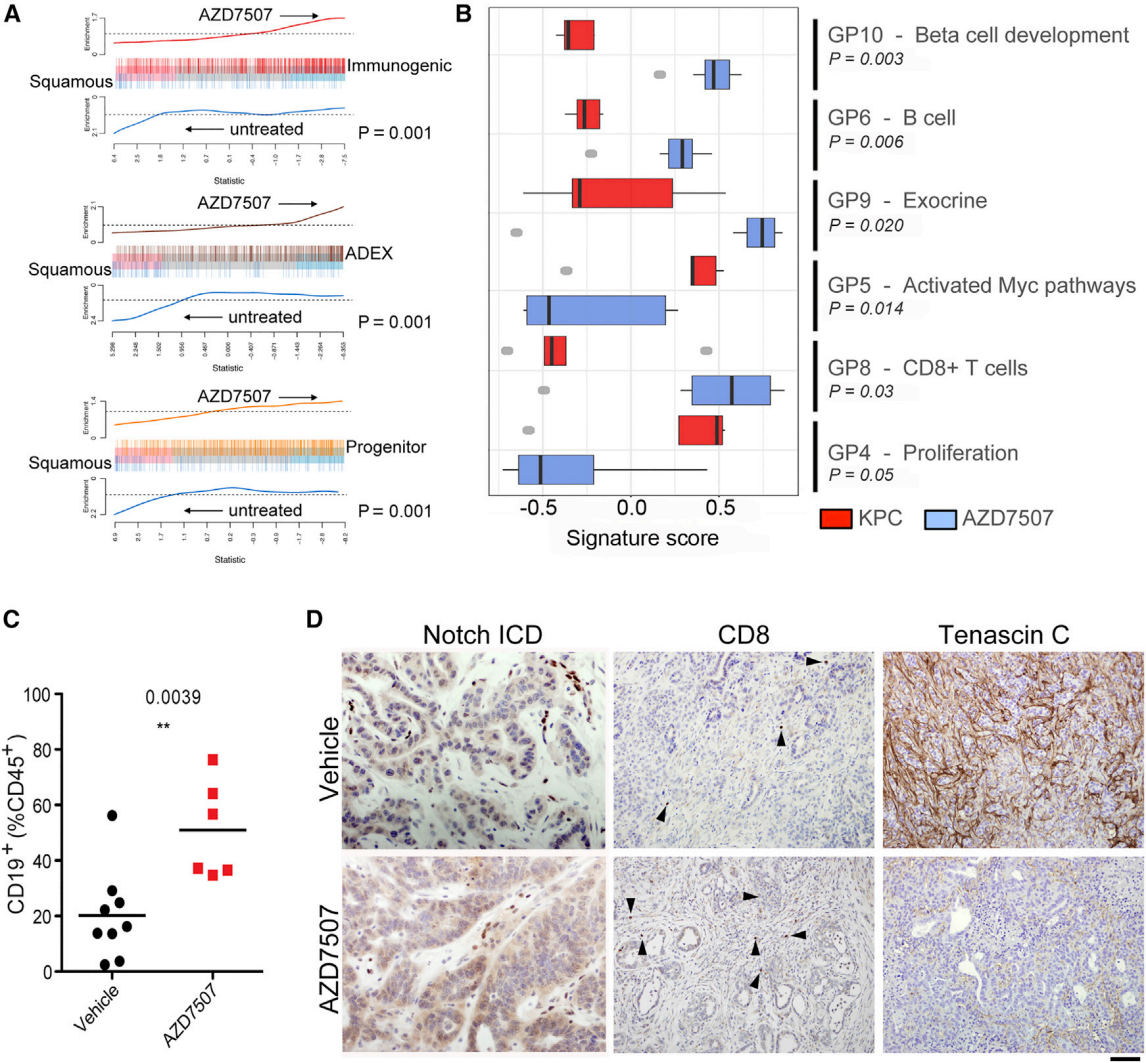
CSF1R Inhibition Results in Significant Reprogramming of Gene Expression Profiles in KPC Mice (A) Barcode plots showing strong enrichment of PDAC class signatures in either control KPC or KPC mice treated with AZD7507. Vertical bars represent signature genes, and lines represent relative signature enrichment. For example, in the top panel, red vertical bars represent genes significantly expressed in the Immunogenic class that are enriched in AZD7507-treated KPC tumors. Enrichment is indicated by an ascending line. p < 0.001 in all cases, n ≥ 5. (B) Boxplots showing the relative enrichment of the indicated gene programs (GP) in AZD7507-treated versus untreated tumor-bearing KPC mice. Boxplots are annotated by a Kruskal-Wallis p value, n ≥ 5. (C) Flow cytometry analysis of CD19^+^ B cells subsets in tumors of mice treated with vehicle (n = 9) or AZD7507 (n = 6). (D) IHC for Notch ICD, CD8 (arrows indicate positive cells), and Tenascin C confirms upregulation of ADEX and Immunogenic signatures and downregulation of Squamous signature. Scale bar, 200 μM. See also Figure S5.

Given the dramatic effect of CSF1R inhibition as a single agent compared to our previous work on CXCR2 inhibition where efficacy in late-stage tumors was only observed in combination with anti-checkpoint inhibition (Steele et al., 2016), we wanted to compare the effect of these inhibitors on tumor gene expression. We focused on the gene expression signatures that define the four recently identified human PDAC subtypes, namely, the “Squamous” subtype, associated with poor outcome, “pancreatic progenitor,” “immunogenic,” and aberrantly differentiated endocrine exocrine, or “ADEX” (Bailey et al., 2016). When global gene expression was examined in our treated KPC tumors, CSF1R inhibition caused a dramatic increase in the “ADEX” and “Immunogenic signatures” and a decrease in the squamous signature (Figure 5A). When specific gene programs were examined, we observed a significantly increased expression of immunogenic programs relating to CD8^+^ T cells, and B cells (Figures 5B and 5C) supporting our earlier findings (Figures 4H and S4F). There were also significant increases in gene programs associated with β-cell development and exocrine pancreas explaining the switch to ADEX subtype. Again, there was a marked impact on malignant epithelial cell gene programs, with a clear reduction in proliferation and programs associated with MYC. Importantly, we confirmed changes in individual components of the gene programs (Figure S5A) by IHC in treated tumors, for Notch activation (GP9 -Exocrine), CD8^+^ T cells (GP8), Tenascin C loss (GP5) (Figure 5D), and CPA1 (GP9) (Figure S5B). The finding of specific genes associated with the ADEX signature in tumor cells strongly suggest the ADEX subtype cannot be explained simply by contaminating acinar and islet tissue, or ADM surrounding tumors.

**Figure 6:**
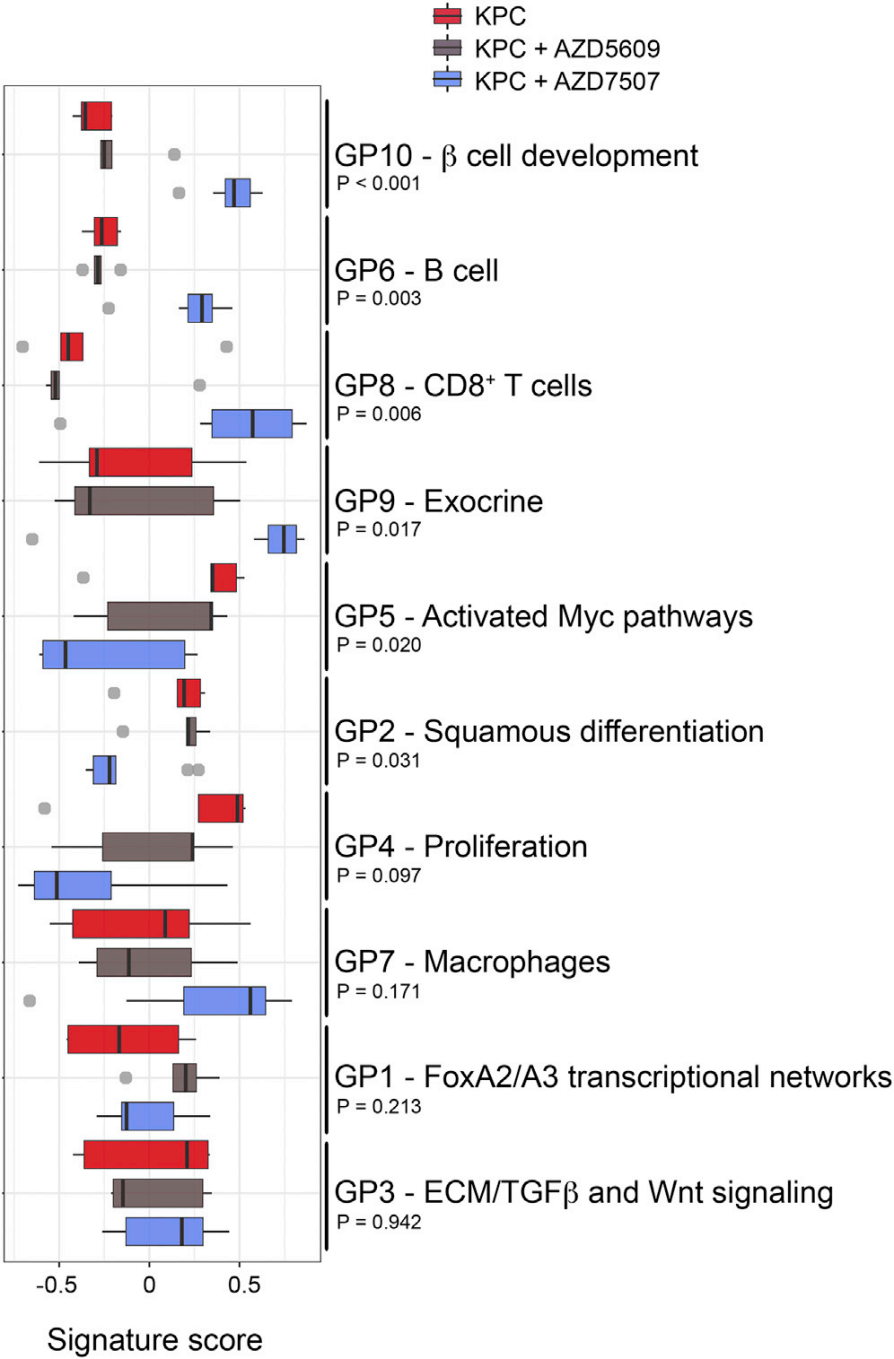
Inhibition of CSF1R Is Not Equivalent to CXCR2 Inhibition Boxplots showing the relative enrichment of the indicated gene programs (GP) stratified by treatment as indicated. Boxplots are annotated by a Kruskal-Wallis p value, n ≥ 5. See also Figure S6.

We then compared the effect of CSF1R inhibition on global gene expression programs in KPC tumors, with the effects of CXCR2 inhibition. CXCR2 inhibition, which, as we previously showed, primarily deleted neutrophils and MDSCs in KPC tumors (Steele et al., 2016), also caused an increase in immunogenic gene programs, but instead of a switch to the ADEX signatures there was a switch to “progenitor” gene programs (Figure 6). The contrasting effects of CXCR2 inhibition versus CSF1R inhibition were also evident within the gene programs, with marked differential expression of genes between the 2 different inhibitions (Figure 6).

We finally analyzed whether genetic loss of CCR2, which is required for myeloid cell recruitment, would recapitulate the phenotype of CSF1R inhibition in the KPC model. However, genetic deletion of CCR2 (myeloid cell) had no impact on primary tumor formation on the KPC model (Figure S6).

## Discussion

PDAC is a deadly disease characterized by desmoplastic stroma. Here, we show that by removing one element of this stroma, CSF1R positive macrophages, many of the elements of the tumor stroma can be changed in a way that is beneficial for therapy. The tumor stroma is switched away from an immunosuppressive environment resulting in a marked increase in CD8^+^ effector cells, while markers of a stiff environment were reduced with changes in collagen (both amount and complexity) and hypoxia (another marker of poor prognosis in PDAC). These alterations, alongside the cytokines and growth factors released by macrophages that will be lacking following CSF1R inhibition, result in reduced tumor cell proliferation and changes in a number of tumor cell intrinsic pathways (MYC, metabolism). This means that, of all the myeloid targeting agents used so far, CSF1R looks the most promising as a single agent, at least in this mouse model of PDAC. This is an important concept given the burgeoning number of potential myeloid target agents that are either ready for, or have entered clinical trials (e.g., CSF1R, IL6, JAK/STAT, CXCR2, and CD40).

A number of subtypes of human PDAC have been defined by global gene expression studies, and we recently found that by inhibiting neutrophil infiltration into KPC PDAC, via CXCR2 targeting, we could observe a gene expression changes that resembled switching of subtype (Steele et al., 2016). There has been much discussion over which signatures come from tumor cells and what might be stromal signatures. Here again, we have found that CSF1R inhibition drives gene expression changes and a subtype switch, although, strikingly, the changes we observe in response to macrophage targeting are markedly different from those we observe when we target neutrophils. Thus, our data would argue that stromal cells drive the characteristics of gene expression in tumor cells.

Recent work by the Jorgenson laboratory has shown how important fibroblast-tumor cell interactions are in driving epithelial gene expression programs (Tape et al., 2016). It is interesting to note here that by altering macrophages we also observe profound differences in markers of cancer-associated fibroblasts (CAFs), with a striking downregulation of αSMA, tenascin C, collagen, and tissue stiffness. Precisely how this occurs will be a focus of future studies, and while we cannot rule out some direct effects of CSF1R inhibition on CAFs, since a subset of these express CSF1R at low levels (Kumar et al., 2017, Öhlund et al., 2017), it will be of interest to see how different elements of the stroma reinforce and amplify signals.

Moreover, we and others have previously shown how important MYC is in PDAC, with a 50% reduction leading to a strong suppression of tumorigenesis (Walz et al., 2014). Given CSF1R inhibition led to a profound reduction in MYC gene programs, it again suggests a very important paracrine role of the stroma in modulating key epithelial driver pathways in epithelial cells. It is important to note that we also have seen clear expression of ADEX gene programs within epithelial tumor cells, which argues against the concept that ADEX is a product of contaminating pancreas.

One interesting open question is the differential dependence on macrophages in the different human subtypes of PDAC. As macrophage density is high in both the squamous/mesenchymal and the immunogenic subtypes (which have a different prognosis), it will be of interest to study in more detail their respective macrophage phenotypes. Recent studies have shown a high heterogeneity of macrophages in human cancer, and there is a large body of work showing that macrophages have many different states (e.g., early studies suggesting M1 V M2 macrophages) and functions/phenotype (Ostuni et al., 2015). Our data here show that macrophages in the KPC model, which aligns with the squamous subtype, express high levels of PD-L1, and this may in part explain the reversal of immune suppression following CSF1R inhibition. It is tempting to suggest macrophages in the human immunogenic subtype may be less immunosuppressive, and therefore more work characterizing macrophage heterogeneity and function in human PDAC is required (Denardo et al., 2011).

There are important differences between the effects we observe here following CSF1R inhibition, and those we found previously when targeting neutrophils via CXCR2 (Steele et al., 2016). Here, we show that CSF1R inhibition produces both a vastly different phenotype but also different mechanistic consequences in terms of inhibiting growth of the primary tumor and relieving T cell suppression. Therefore, delineating the differences between inhibition of different myeloid cells is very important. Two recent studies confirm and extend our observations on the very different mechanisms and consequences of inhibiting different myeloid cell populations. In orthotopic PDAC transplant models, inhibition of CXCR2 alongside CCR2 improved chemotherapeutic efficacy due to a compensatory increase in CXCR2 or CCR2 positive cells if a single inhibitor was used (Nywening et al., 2017). In a series of syngeneic lines, Kumar et al. (2017) showed that CSF1R inhibition led to increased production of granulocyte/neutrophil chemokines from fibroblasts, provoking their recruitment. Combining inhibition of CXCR2 with CSF1R stopped the recruitment of these neutrophil/granulocytes and improved efficacy. It is interesting to note here that in the autochthonous KPC model we did not see such a dramatic increase in CXCR2 positive cells on treatment with the CSF1R inhibitor. However, much further work is required in both the scheduling and combinations of neutrophil, macrophage, and checkpoint inhibition ahead of clinical trials.

In summary, we have identified a critical and distinct role for CSF1R positive macrophages in PDAC that should underpin future clinical trials. Taken together with our previous work, these preclinical studies suggest that sequential scheduling of treatments that target different myeloid cell populations may be of benefit in patients with advanced pancreatic cancer.

## Experimental Procedures

### Pancreatic Cancer Tissue Microarray

Tissue microarrays containing 5 cores of resected PDAC from each of 79 adult patients were obtained from Greater Glasgow and Clyde NHS Biorepository. Tissue was collected prospectively with local ethical approval (West of Scotland Research Ethics Service REC reference number 09/S0704/42) and fully informed consent. Only histologically proven PDACs with complete clinicopathological, follow-up, and recurrence data were included. Kaplan-Meier survival analysis was used to analyze survival from time of surgery, and log-rank tests were used to compare length of survival between curves. Statistical significance was set at a p value of <0.05. All statistical analyses were performed using SPSS version 19.0 (version 19.0. Armonk, NY: IBM) Following IHC, expression was scored using HALO software, either as percentage positive staining (CSF1R) or as percentage of positive cells (CD3).

### Human Pancreatic Tissue and Plasma

All tissues were collected with ethical approval and fully informed consent. Dr. Anne Schultheis (University Hospital Cologne, Germany) kindly provided human pancreas tissue slides.

Blood samples were collected under ethical approval (REC05/Q0408/65), and written informed consent was obtained from all patients. Patients were selected that had a confirmed diagnosis of unresectable locally advanced or metastatic stage III–IV PDAC. Blood was collected in anti-coagulant-treated EDTA vacutainer tubes. Whole blood was transferred to falcon tubes and centrifuged. The plasma supernatant was collected and aliquot into 1.5-mL Eppendorf tubes.

### IHC and Immunofluorescence

IHC was performed on formalin-fixed paraffin-embedded pancreatic tissue using standard protocols, whereas immunofluorescence was performed on formalin-fixed paraffin-embedded (human) and frozen (mouse) pancreatic tissue. For immunofluorescence, slides were mounted using Prolong Gold antifade reagent with DAPI. For antibodies, see Supplemental Experimental Procedures.

### RNAscope

*In situ* detection of PD-L1 transcripts in formalin-fixed paraffin-embedded (FFPE) mouse PDAC samples was performed using a PD-L1-specific RNAscope assay (Advanced Cell Diagnostics) according to the manufacturer’s protocol.

### Animal Experiments

All animal experiments were performed under Home Office license and approved by the University of Glasgow Animal Welfare and Ethical Review Board. Mice were maintained in conventional cages and given access to standard diet and water *ad libitum*. Female SCID mice aged 6–8 weeks were obtained from Taconic (Germantown, PA). KPC mice, first described by Hingorani et al. (2005), were bred in house on a mixed background. Mice were genotyped by Transnetyx (Cordoba, TN, USA). Mice were monitored at least 3 times weekly and culled when exhibiting symptoms of PDAC. For short-term drug studies, pancreatic malignancy was confirmed by abdominal palpation. For drug treatments, adult mice of both sexes were randomly assigned to cohorts. See Supplemental Experimental Procedures for more information.

### Cell Culture

KPC-derived murine pancreatic cancer cell line was obtained from Prof David Tuveson (Cold Spring Harbor, USA). Human PDAC cell lines PANC-1 and MIA PaCa-2 were purchased from the American Type Culture Collection (ATCC), and both were derived from male pancreatic duct tumors (Deer et al., 2010). PDAC cell lines were maintained in complete culture medium with DMEM, 10% v/v fetal bovine serum (FBS), and 1% v/v penicillin/streptomycin under sterile conditions at 37°C and 5% v/v CO_2_ atmosphere. For information on bone-marrow-derived macrophages and cell-culture assays, see Supplemental Experimental Procedures.

### CSF1-R *In Vitro* Enzyme Assay

Activity of CSF1R kinase domain (aa 568–912 GeneBank ID NM_005211) was determined using an AlphaScreen assay (PerkinElmer, MA) measuring phosphorylation of a biotinylated polyGT peptide (Cisbio).

### Western Blotting

Electrophoresis was performed using standard protocols. For more information, see Supplemental Experimental Procedures.

### Flow Cytometry

Pancreas was collected in ice-cold PBS and washed in Hank’s balanced salt solution (HBSS) solution before mincing using scalpels. The pieces were then incubated in 2 mg/mL collagenase (Sigma) in HBSS with 50 μg/mL DNase (Sigma) for 20 min at 37ºC in a shaker. The almost-dissolved pieces were then passed through a 70-μm cell strainer and resuspended in flow cytometry buffer and cells counted. Antibody labeling and flow cytometry were carried out using standard protocols. For more information, see Supplemental Experimental Procedures.

### RNA Extraction

Portions of murine pancreatic tumors were frozen in RNAlater (QIAGEN) solution until required. RNA extraction was performed using QIAGEN RNeasy Plus Mini Kit (QIAGEN) and homogenized in a Precellys with ceramic beads (Stretton scientific). DNA was removed with Turbo DNA-*free* Kit (Applied Biosystems).

In short-term treatment studies, 25 mg of frozen pancreatic tissue was disrupted and homogenized by placing them into gentleMACS M tubes with 1 mL of 4% B-mercaptoethanol (Sigma) in RLT buffer. Samples were processed using the gentleMACS dissociator set to the RNA-02 program. RNA integrity was determined using Agilent 2100 Bioanalyzer using the RNA 6000 Nano assay (Agilent Technology) according to the manufacturer’s protocol.

### Affymetrix Analysis

Affymetrix was performed using the GeneChip Hybridization, Wash, and Stain Kit, GeneChip 3′ *in vitro* transcription (IVT) Express Kit, and the GeneChip Mouse Genome 430 2.0 Array according to the manufacturer’s instructions (GeneChip 3′ IVT Express Kit, User manual P/N 702646 Rev 8).

### Second Harmonic Generation Analysis

Analysis of the deposition and higher-order structure of stromal collagen in tumors was examined through analysis of second harmonic resonance produced as a result of multiphoton microscopy. For more information, see Supplemental Experimental Procedures.

### RNA-Seq Analysis

Sequencing reads were mapped to the mouse mm10 genome using the RNA-seq pipeline implemented by the bcbio-nextgen project (https://bcbio-nextgen.readthedocs.io/en/latest/). For more information, see Supplemental Experimental Procedures.

### Statistical Analysis

Kaplan-Meier survival analysis was performed and survival between cohorts compared using log-rank tests (n = number of mice). Assessment of differences in average counts between different mice was performed using non-parametric Mann-Whitney testing and unpaired t tests (n = number of mice). Analyses were performed using GraphPad Prism software (version 5.0).

## Data and Software Availability

The accession number for the RNA-seq data reported in this paper is ArrayExpress: E-MTAB-6620 (https://www.ebi.ac.uk/arrayexpress).

## Supplemental Information

Document S1. Supplemental Experimental Procedures, Figures S1–S6, and Table S1

Document S2. Article plus Supplemental Information
