# Supplementary material for: CSF1R+ Macrophages Sustain Pancreatic Tumor Growth through T Cell Suppression and Maintenance of Key Gene Programs that Define the Squamous Subtype
Source: Cell Rep. 2018 May 2;23(5):1448–60. doi: 10.1016/j.celrep.2018.03.131 (PMC5946718; doi:10.1016/j.celrep.2018.03.131)
Supplement: Document S1. Supplemental Experimental Procedures, Figures S1–S6, and Table S1 [file mmc1.pdf]

Cell Reports, Volume *23*

Supplemental Information

CSF1R^{+} Macrophages Sustain Pancreatic Tumor Growth through T Cell Suppression and Maintenance of Key Gene Programs that Define the Squamous Subtype

Juliana B. Candido, Jennifer P. Morton, Peter Bailey, Andrew D. Campbell, Saadia A. Karim, Thomas Jamieson, Laura Lapienyte, Aarthi Gopinathan, William Clark, Ewan J. McGhee, Jun Wang, Monica Escorcio-Correia, Raphael Zollinger, Rozita Roshani, Lisa Drew, Loveena Rishi, Rebecca Arkell, T.R. Jeffry Evans, Colin Nixon, Duncan I. Jodrell, Robert W. Wilkinson, Andrew V. Biankin, Simon T. Barry, Frances R. Balkwill, and Owen J. Sansom

Figure S1

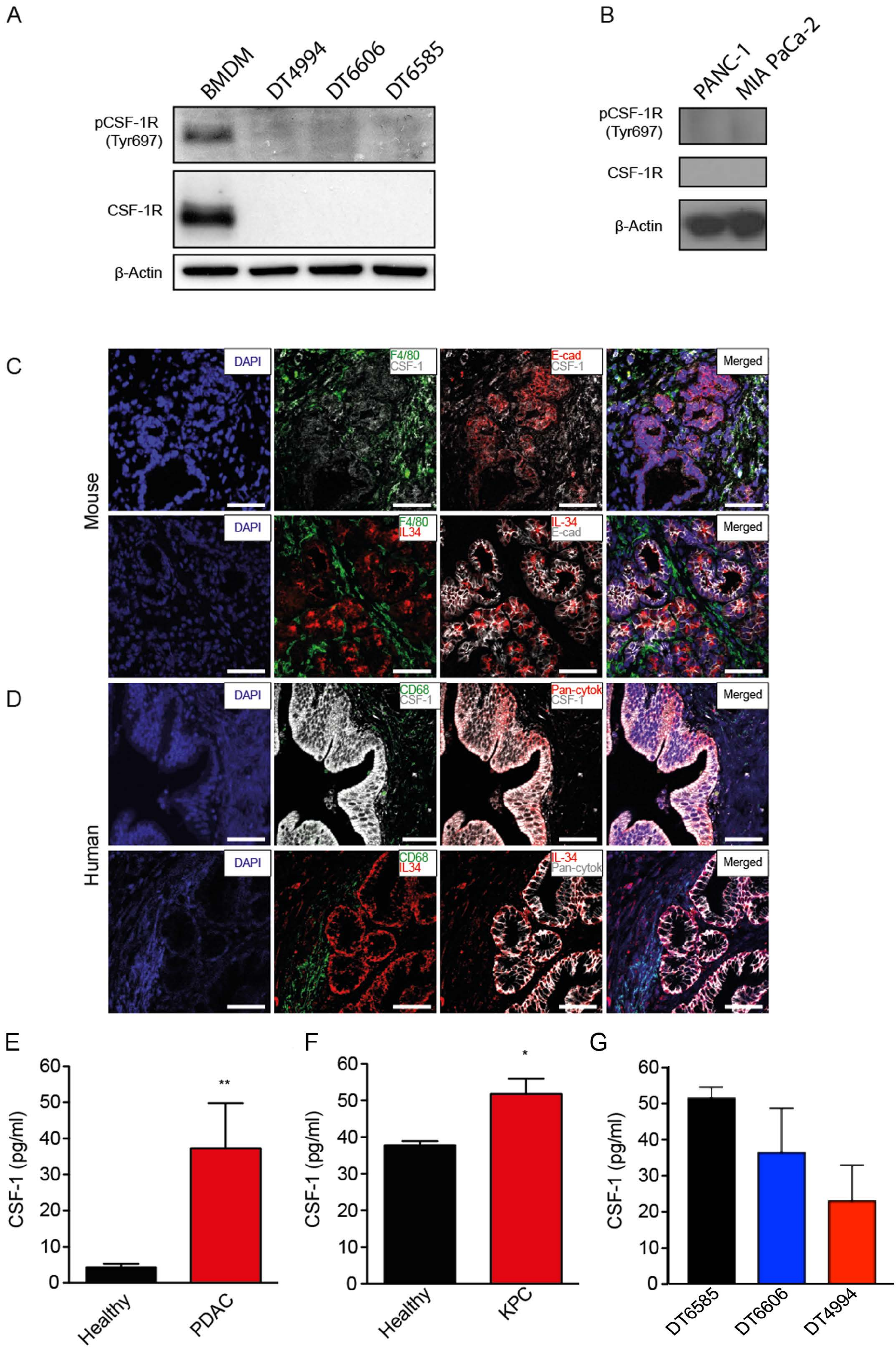

Figure S1 related to Figure 1

A) Western blot analysis of murine BMDM and murine-derived pancreatic cancer cell lines (DT4994, DT6606 and DT6585) for pCSF-1R (Tyr697) and total CSF-1R. β-actin was used as loading control. B) Protein lysate from human pancreatic cell lines (PANC-1 and MIAPaCa-2) were analyzed for the phosphorylation of CSF-1R (Tyr697), total CSF-1R and β-actin by Western Blot. C) Tissue sections from mouse pancreas were stained against F4/80 (green) and with CSF-1 (grey) or IL-34 (red) and against E-cadherin (red for CSF-1, grey for IL-34 and counterstained with DAPI (blue). Scale bar 100 µm. D) Pancreatic tissue sections of patients diagnosed with PDAC were stained with antibodies against CD68 (green), CSF-1 (grey) or IL-34 (red) and pan-cytokeratin (red for CSF-1, grey for IL-34) and counterstained with DAPI (blue). Scale bar: 50 µm. E) Human plasma was measured for CSF-1 levels using the MSD electroluminescence assay from age-matched healthy individuals and PDAC patients. F) Murine plasma was measured for CSF-1 levels and analyzed via MSD assay. The levels of cytokines measured in the different groups were compared using the Mann-Whitney U test. Data show mean of all values of from at least five samples per group. *P< 0.05, **P< 0.01 and ***P< 0.001, n>5. G) CSF-1 production by 3 murine-derived KPC tumour cell lines was measured by ELISA.

Figure S2

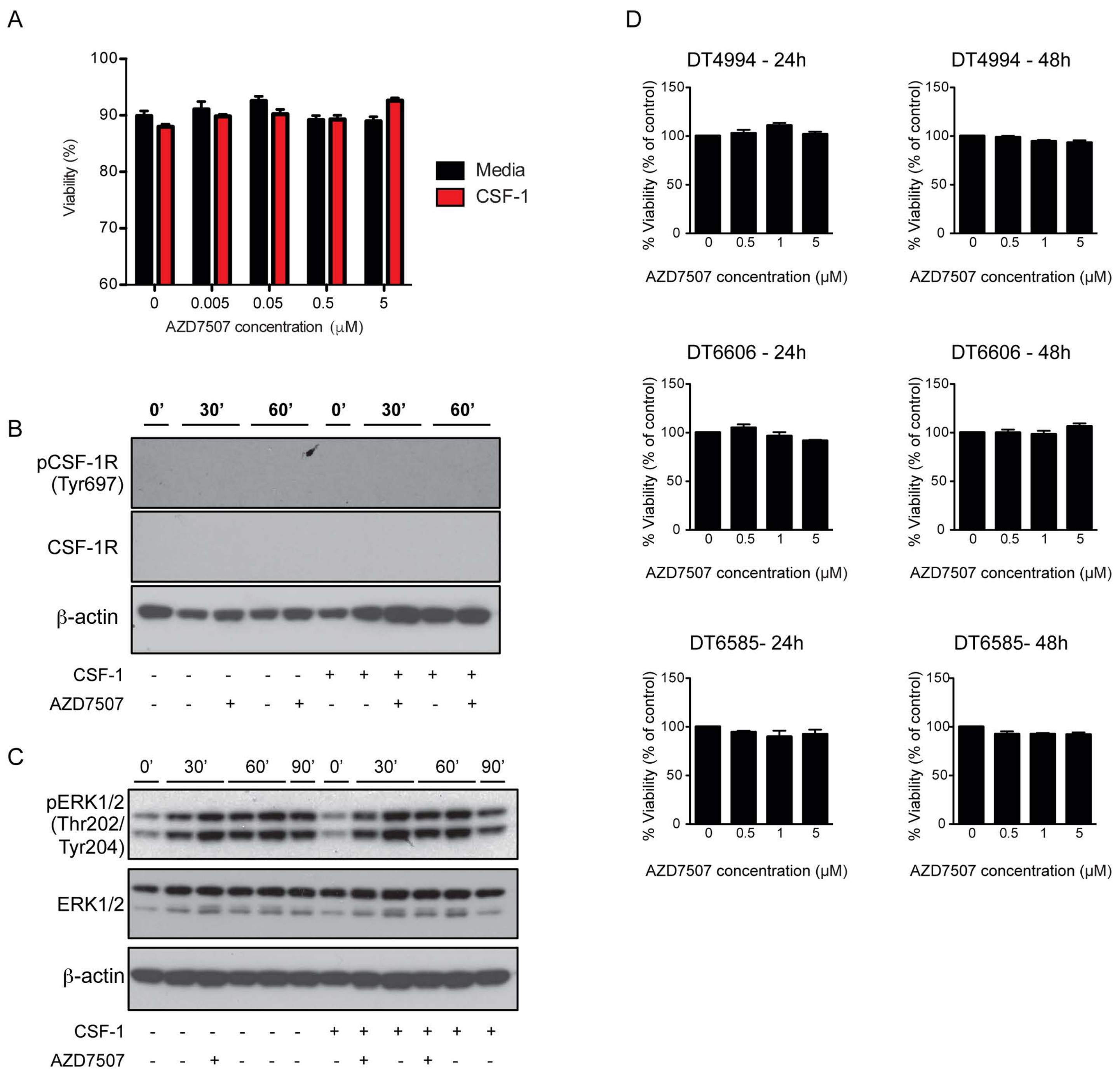

Figure S2 related to Figure 2

A) BMDM were cultured with or without CSF-1 for 24h in the presence or absence of different concentrations of AZD7507. Culture supernatants were assessed for membrane integrity by measuring the release of LDH. Results are shown as percentage of viable cells as compared to the positive control. Data are represented as mean +SEM for n=6. B-C) TB32047 PDAC cells were treated with or without CSF-1 in the presence or absence of 5 μM AZD7507 and protein analyzed at indicated time points. Protein lysates were probed for B) pCSF-1R (Tyr697), total CSF-1R and β-actin, or C) phosphorylated-ERK1/2 (pERK1/2) (Thr202/Tyr204), total ERK1/2 and β-actin. D) Using three different cell lines, either media or AZD7507 inhibitor was added at 0 (control media), 0.5, 1 or 5 μM for 24 and 48 hours and cell viability assessed by the WST-1 colorimetric absorbance assay. The bar graph shows the percentage viability of AZD7507 treated cells when compared to cells treated with vehicle. Data are represented as mean + SEM for n=5.

Figure S3

A

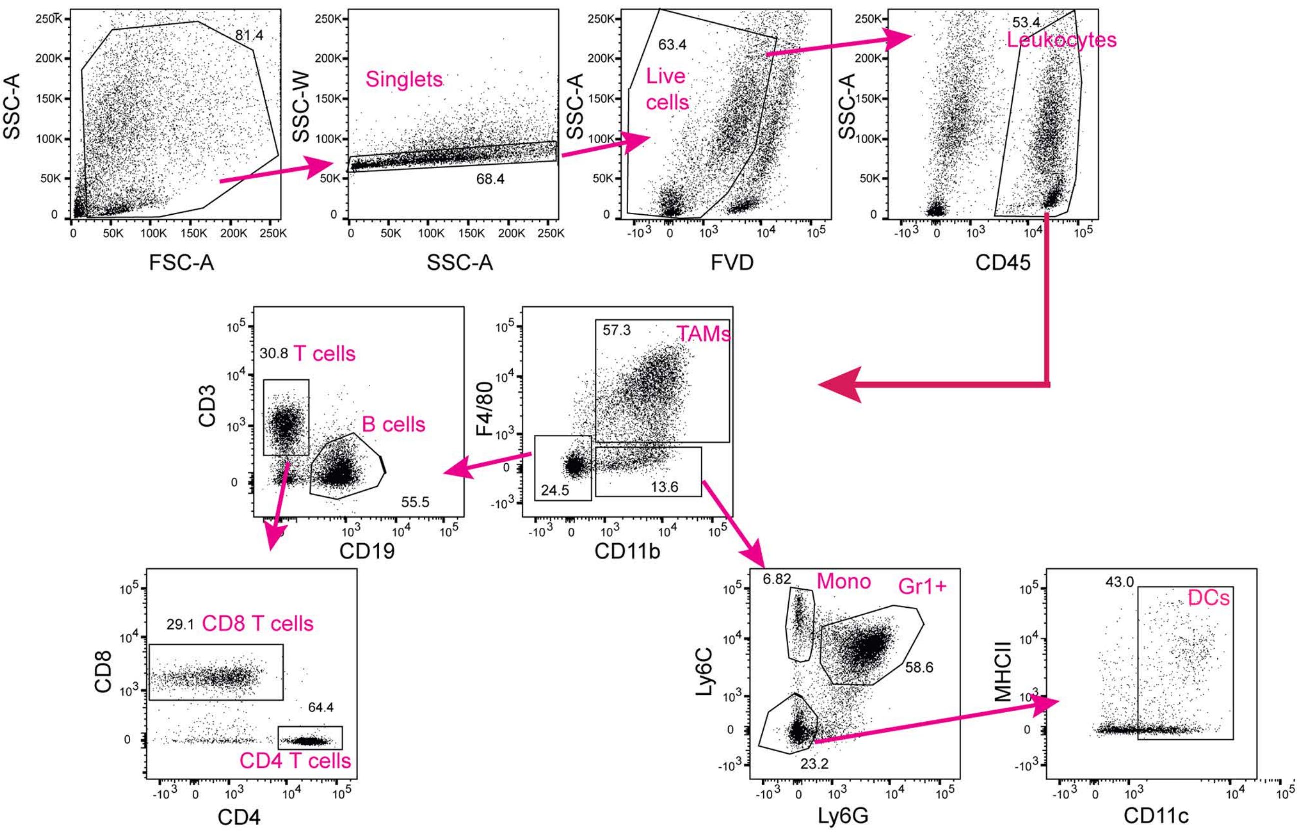

B

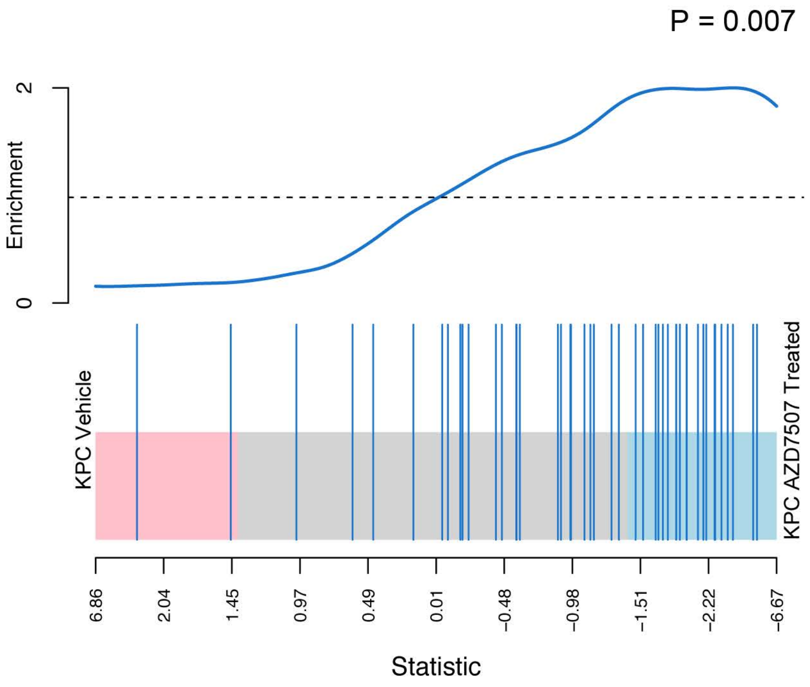

Figure S3 related to Figure 3

A) Gating strategy and representative gates used for leukocyte populations in the tumor of KPC mice by flow cytometry. Total tumor cell suspensions were prepared after digestion with collagenase and stained with CD45, CD11b, F4/80, Ly6G, Ly6C, CD11c, CD3, CD19, CD4 and CD8 fluorescence antibodies. Debris, doublets and dead cells were excluded from the analysis. Tumor associated macrophages (TAMs) are defined as: CD45+CD11b+F4/80+ cells, Monocytes as CD45+CD11b+F4/80-Ly6G-Ly6C+, Gr1+ cells as CD45+CD11b+F4/80-Ly6G+Ly6C+, Dendritic cells (DCs) as CD45+CD11b+F4/80-Ly6G-Ly6C-CD11c+, B cells as CD45+CD11b-F4/80-CD3-CD19+, CD4 T cell as CD45+CD11b-F4/80-CD19-CD3+CD4+ and CD8 T cells as CD45+CD11b-F4/80-CD19-CD3+CD8+. B) Barcode plot showing enrichment of the M1 macrophage signature in KPC mice treated with AZD7507. Vertical bars represent signature genes and lines represent relative signature enrichment. Enrichment is indicated by an ascending line. P = 0.001.

Figure S4

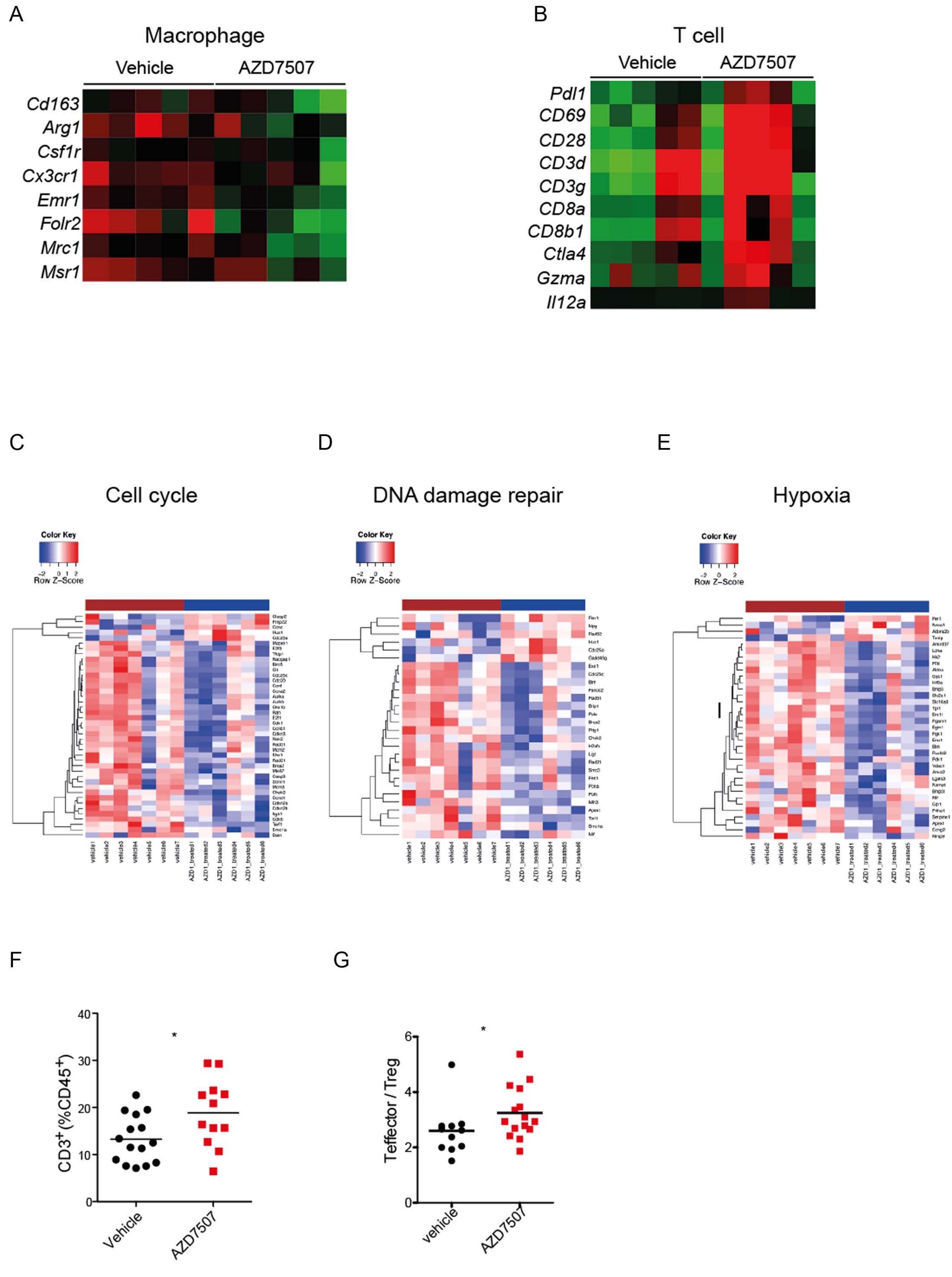

Figure S4 related to Figure 4

A-B) Heat map representation of selected gene expression profiles related to A) macrophages and B) T cells from tumors of vehicle treated and AZD7507 treated KPC mice after 5 days were analyzed using the Affymetrix platform. Data were from 5 biological replicates for tumor lysates. Genes with the greatest differences in red: high and green: low. C-E) Differential gene expression analysis using RNA sequencing. Clustering heat map of all 770 differentially expressed genes using FDR < 0.05 between tumors of vehicle treated and AZD7507 treated KPC mice after 14 days. Selected Gene Ontology categories and their enrichment for genes related to C) cell cycle, D) DNA damage repair and E) hypoxia. Flow cytometry analysis of the frequency of F) CD3+ T cells and G) Teffector/Treg ratio in the tumor of mice treated with either vehicle or AZD7507. P values were calculated by Mann-Whitney U test (*P< 0.05, **P< 0.01 and ***P< 0.001), mean +/- s.e.m.

Figure S5

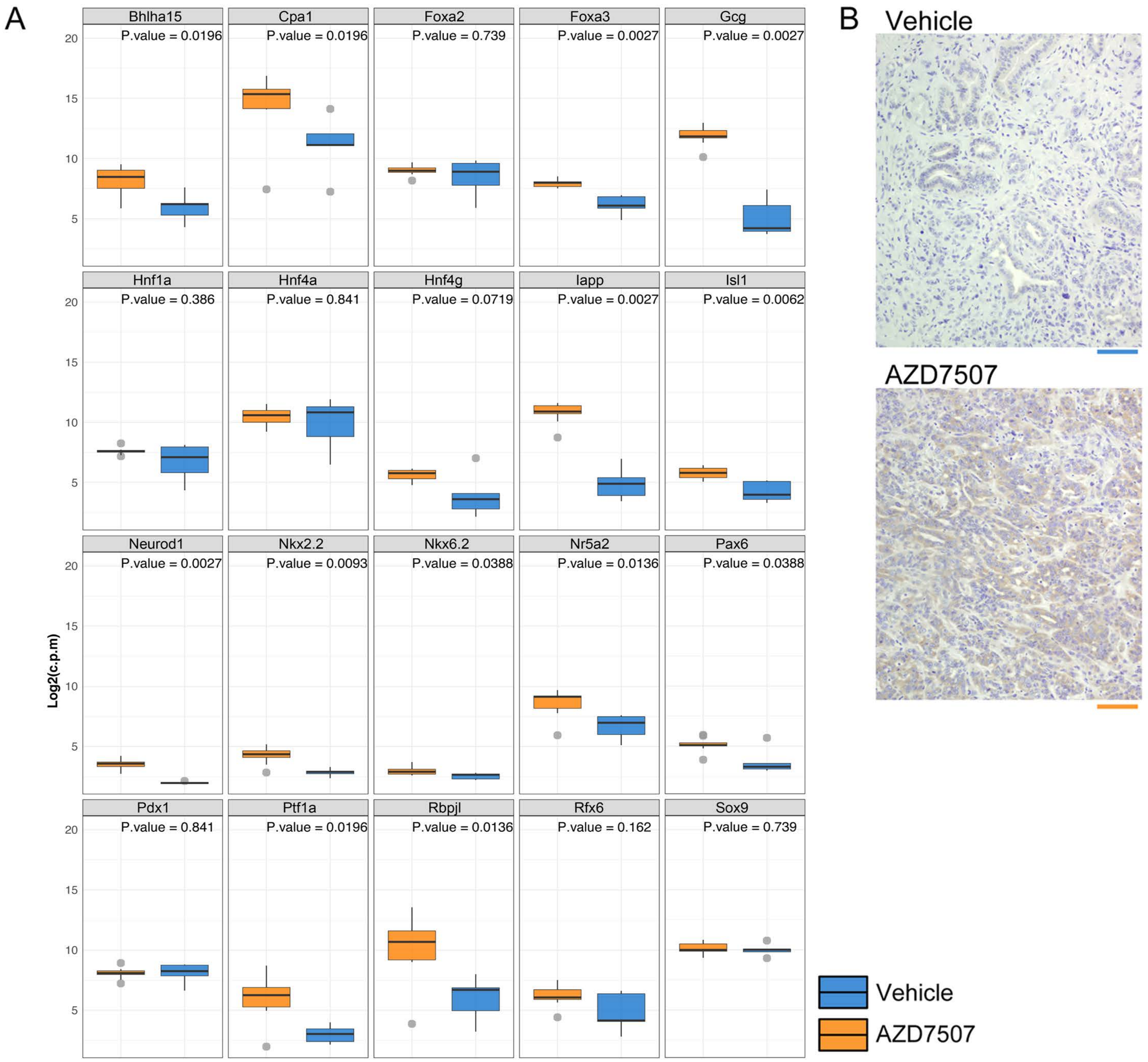

Figure S5 related to Figure 5

Inhibition of CSF1R is not equivalent to CXCR2 inhibition. A) Boxplots showing the expression of selected genes involved in pancreatic development and maintenance in AZD7507 treated vs untreated tumor-bearing KPC mice. Boxplots are annotated by a Kruskall-Wallis P value. B) IHC for Carboxypeptidase1 (Cpa1) in control vs AZD7507-treated tumor-bearing KPC mice. Scale bar = 200 µM.

Figure S6

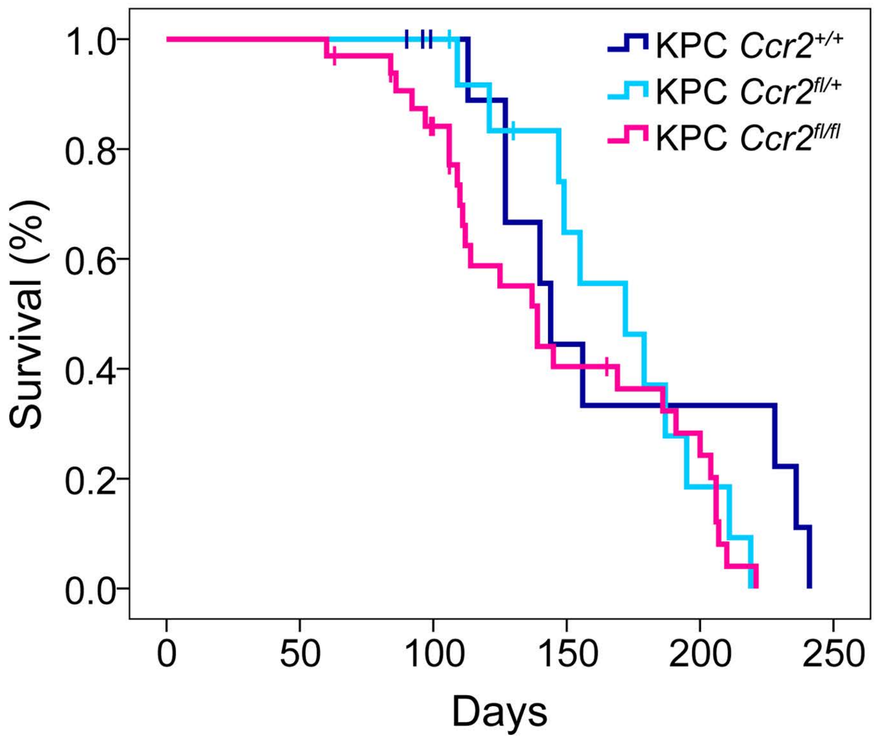

Figure S6 related to Figure 6

Genetic deletion of CCR2 has no effect on tumor formation in the KPC model. Kaplan-Meier analysis of survival of KPC (n=12), KPC Ccr2fl/+ (n=13) and KPC Ccr2fl/fl mice. Mice sacrificed due to pathologies other than PDAC are censored and indicated by ticks.

Supplementary Table 1

AZD7507	% Control	AZD7507	% Control
Csf-1r	-1	PKCζ	97
cKit	10	SRPK1	97
ARK5	22	CLK3	98
Flt4	36	CSK	98
Rsk1	55	p70S6K	98
GSK3β	58	PhKγ2	98
Axl	63	WNK3	98
CHK1	73	ASK1	99
FAK	73	BTK	99
PDGFRα	75	DDR2	99
CK1γ1	76	Fgr	99
PKD2	82	JAK2	99
MRCKα	83	LIMK1	99
LOK	84	NLK	99
BrSK2	85	FGFR1	100
JNK1α1	86	GRK5	100
KDR	86	MLK1	100
MELK	87	MSSK1	101
PKA	88	EphA7	103
c-RAF	89	MINK	103
TSSK2	89	CK2	104
IGF-1R	90	EGFR	104
STK33	90	Itk	105
ALK4	91	LKB1	105
Met	91	MAPK1	105
VRK2	91	DK2/cyclin	107
DYRK2	92	Abl	110
Ret	92	DAPK1	110
SIK	92	MEK1	110
Aurora-A	93	Mnk2	110
Pim-2	94	MAPKAP-K	111
IRAK4	95	NEK7	112
ALK	96	EphB4	113
Flt3	96	SAPK2a	113
IKKα	96	Tie2	115
P1K3	96	PKBβ	120
TrkA	97	TAK1	120
CK2α2	97	NEK2	121
cSRC	97	HIPK1	125
PAK2	97	MKK4	188
PDK1	97		

Supplementary Table 1 related to Figure 2

Kinase profiler data for AZD7507 at 1μM against 81 kinases.

SUPPLEMENTAL METHODS

Immunohistochemistry and Immunofluorescence

The following antibodies were used:

Protein	Clone	Conc.	Supplier
CD68	PG-M1	1/50	Dako
CD68-FITC	Y1/82A	1/50	eBioscience
F4/80	A3-1	1/100	Bio-Rad
F4/80-AlexaFluor488	BM8	1/50	eBioscience
E-Cadherin-AlexaFluor647	DECMA-1	1/100	eBioscience
CSF1	EP1179Y	1/50	Abcam
CSF-1R mouse		1/100	Cell Signaling Technology
CSF-1R human	SP211	1/100	Abcam
CD8	4SM15	1/50	eBioscience 14-0808-82
alpha-SMA	clone 1A4	1/500	Sigma-Aldrich
Notch ICD	D3B8	1/50	Cell Signaling Technology
IL-34		1/50	Abcam
Pan-cytokeratin	PCK-26	1/300	Abcam
Carboxypeptidase A	EPR2086	1/500	Abcam
Tenascin C	EPR4219	1/500	Abcam

In vivo treatment experiments

For drug treatments, adult mice of both sexes were randomly assigned to cohorts. Pancreatic malignancy was confirmed by abdominal palpation. For tumor measurement, high-resolution ultrasound imaging was performed using the Vevo3100 System, and tumors measured from

two dimensional images at the maximal dimensions of the tumor. Anesthesia was induced and maintained throughout the procedure with a mixture of isoflurane and medical air. Treatments used were: CXCR2 small molecule inhibitor (AstraZeneca, AZD5069) at 100mg/Kg p.o. twice daily; vehicle (0.1 % Tween80/0.5 % methylcellulose) p.o. twice daily and CSF-1R small molecule inhibitor (AstraZeneca, AZD7507) at 100mg/Kg p.o. twice daily; vehicle p.o. twice daily.

Female SCID mice aged 6 to 8 weeks were obtained from Taconic (Germantown, PA) and mice were injected with $8x10^6$ MDA-MB-231 estrogen receptor negative cells into the mammary fat pad and grown until tumors were an average volume of 100 mm^3. Mice were treated twice daily with vehicle, 10 mg/kg, 30 mg/kg and 100 mg/kg of AZD7507 for 20 days.

Cell culture experiments

Bone Marrow Derived Macrophages

Tibias and femurs were harvested from healthy mice. Under sterile conditions bones were held with sterile forceps and a 27G needle syringe containing 10 ml of PBS was used to flush the bone marrow. Red blood cell lysis was used and single cell suspension was passed through a 70 µm cell strainer. Cells were resuspended in complete medium and plated in a 140 mm bacterial Petri dish containing 20 ng/ml of recombinant CSF-1 and incubated for 6 days.

Lactate Dehydrogenase Assay

LDH activity in culture supernatants was established using the fluorescence-based CytoTox-ONE kit (Promega). Bone marrow derived macrophages (BMDM) were plated in a white 96-well in complete medium or complete medium with 20 ng/ml of CSF-1 and in the presence of either AZD7507 concentrations or vehicle for 24 hours.

Apoptosis Assay

500,000 bone marrow cells were cultured with 20 ng/ml CSF-1 complete medium in the presence or absence of AZD7507 at difference concentrations and apoptotic cells were detected at day 7. The apoptotic cells were determined by APC-labeled Annexin V (eBioscience), CSF1-R-BV421 (eBioscience) and fixable viability dye (FVD)-e506 (eBioscience) staining and detected by flow cytometry. The population of apoptotic bone marrow cells was identified as CSF-1R+/Annexin V+/FVD-.

WST-1 Assay

Cell viability was measured using a WST-1 assay (Roche). Briefly, 10,000 PDAC cells were cultured in 96-well flat-bottomed plates with 20 ng/ml CSF-1 in the presence or absence of AZD7507 at difference concentrations and absorbance measured after 3 and 7 days.

Western Blotting

Electrophoresis was performed using the Invitrogen NuPAGE® System. Samples were prepared by adding 15 µg of protein according to the manufacturer's protocol. Small to medium proteins (molecular weight: 20-100 KDa) were separated in NuPAGE® 4-12 % Bis-Tris gels. Large proteins (above 100 KDa molecular weight) were separated in NuPAGE® 3-8 % Tris-Acetate gels. Resolved proteins were transferred using Invitrolon™ PVDF/Filter Paper Sandwiches (Invitrogen) and XCell II™ Blot Module system (Invitrogen). Immunodetection was performed by adding a substrate for the HRP (Amersham ECL™ Western Blotting Detection Reagents) followed by an exposure onto Super RX autoradiography films. The following antibodies were used phosphor-CSF-1R (Tyr699), CSF-1R, phosphor-ERK1/2 (Thr202/Tyr204) and ERK1/2 all from Cell Signaling Technology. B-actin was purchased from Sigma-Aldrich.

Flow cytometry

Pancreas was collected in ice-cold PBS and washed in HBSS solution before mincing using scalpels. The pieces were then incubated in 2mg/ml collagenase (Sigma) in HBSS with 50 µg/ml DNase (Sigma) for 20 minutes at 37C in a shaker. The tissue was then passed through a 70 µm cell strainer and resuspended in flow cytometry buffer and cells counted.

Cells were plated in a 96-well plate (3 million cells/well) and cells resuspended in Fc block (CD16/32, eBioscience), incubated on ice and mastermix of antibodies added. After washing cells were stained with a fixable viability dye-e506 (eBioscience) in PBS. For extracellular only staining, cells were then resuspended in Fluorophix (Biolegend) and cells were washed and resuspended in flow cytometry buffer. For intracellular staining, cells were resuspended in Foxp3 Intracellular staining kit (eBioscience) according to their protocol. Flow cytometric analysis was performed using an LSRFortessa cell analyzer (BD Biosciences) and FACSDiva software Version 6.2. Data were transferred and analyzed using the FlowJo software (Tree Star, Oregon, USA). Antibodies used: CD45 (30-F11), CD3 (145-2C11), CD4 (RM4-5), CD8 (53-6.7), CD11b (M1/70), CD11c (N418), F4/80 (BM8), CD19 (eBio1D3), MHCII (M5/114.15.2) all from Biolegend. Ly6G (RB6-8C5), Ly6C (HK1.4) and Foxp3 (FJK-16s) antibodies were purchased from eBioscience.

Depletion of F4/80- cells from KPC Pancreatic Tumors

Tumors from KPC mice were digested as described above and cells stained with F4/80-PE (eBioscience). F4/80+ cells were positively selected using anti-PE immunomagnetic beads according to the manufacturer's instructions (Miltenyi).

To measure CD8 T cell cytokine production, 96-well round-bottomed plates were pre-coated with anti-CD3e (Biolegend) overnight. Pancreatic tumor cell suspension with or without F4/80+ cells (3:1 ratio) was incubated in the presence of soluble anti-CD28 (Biolegend) for

72h in complete medium. After 72h, cells were incubated with cell stimulation cocktail and transport inhibitors (eBioscience) for 5 hours and cells were stained for flow cytometry analysis using the surface markers: CD45-BV570 (Biolegend), CD3-FITC (eBioscience) and CD8-BV650 (Biolegend). Fixable viability die-e506 was used to distinguish live/dead cells. Intracellular staining for Granzyme B-e450 (eBioscience), Perforin-PE (eBioscience) and IFNg-PE-Cy7 (eBioscience) was performed using the Foxp3/transcription factor staining buffer set from eBioscience according to their instructions.

Cytokine Measurements

Pancreas was collected, weighed and lysis buffer (50mM Tris-HCl with 2mM EDTA, pH7.4) with phosphatase and protease inhibitors was added. Tissues were lysed by gentle MACS Dissociator set using M tubes (Miltenyi Biotec). Using a probe sonicator set at 40% amplitude, the lysate was sonicated on ice. Tubes were placed in a rotator at 4C followed by centrifugation. Supernatant was stored at -80C until further analysis.

Enzyme-Linked Immunosorbent Assay (ELISA): ELISA kit was purchased from R&D and used to measure plasma human M-CSF (DMC00B) according to their instructions.

Meso Scale Discovery (MSD): Prototype Assay using biotinylated mouse M-CSF was used to measure the levels in the plasma according to manufacturer instructions.

Myriad: Samples were sent to Myriad RBM (Austin, TX, USA) for quantitative measurement of cytokine and chemokine analytes (Mouse Inflammation MAP v.10 array) utilizing microsphere-based immune-multiplexing assay on the Luminex platform.

SHG analysis

Analysis of the deposition and higher-order structure of stromal collagen in tumors was examined through analysis of second harmonic resonance produced as a result of multiphoton

microscopy. Collagen second-harmonic images were collected using a LaVision Biotec Trim-scope equipped with a Coherent Chameleon Ti:Sapphire femtosecond pulsed laser. An excitation wavelength of 890 nm was used so that the second-harmonic signal would be generated at a central wavelength of 445 nm and focused to the sample plane by a long working distance 20x (NA = 0.95) water immersion objective. At least 5 x 40 µm z-stacks per sample were generated over a region of 500µm by 500µm, based upon an initial histological identification of PDAC. Maximum projection images of each z-stack were then thresholded to remove background noise, with subsequent grey level co-occurrence matrix (GLCM) analysis of imaged fibrillar collagen performed using an openware ImageJ plugin (https://imagej.nih.gov/ij/plugins/texture.html). Correlation profiles were generated, which describe linear co-dependence of grey levels between pixels at defined comparison distances in either the x or y plane across all images generated from all biological specimens in either group, and double exponential decay curves subsequently fit to data.

RNA Seq Analysis

Sequencing reads were mapped to the mouse mm10 genome using the RNA-seq pipeline implemented by the bcbio-nextgen project (https://bcbio-nextgen.readthedocs.org/en/latest/). Briefly, after quality control and adaptor trimming, reads were aligned to the UCSC mouse mm10 genome build using STAR (Dobin et al., 2013). Counts for known genes were generated using the function featureCounts in the R/Bioconductor package "Rsubread" (Liao et al., 2014). The R/Bioconductor package "DESeq2" was used to identify differentially expressed genes (Love et al., 2014). Barcode plots were generated using the R/Bioconductor package "edgeR" (Law et al., 2014). Gene ontology networks were generated using the ClueGO-CluePedia cytoscape plugin (Bindea et al., 2013). Gene sets representing PDAC classes were obtained from (Bailey et al., 2016) and generated by selecting significantly upregulated genes

in a given class versus all other classes. An adjusted *P* value of 0.01 was used as the cut-off in each case. Gene sets representing macrophage phenotypes M1 and M2 were obtained from (Jablonski et al., 2015). To test whether any of the genes in the relevant gene set were differentially expressed between treatment and control we performed *roast* (Rotation Gene Set Tests) analysis as implemented by the R package *limma* (Ritchie et al., 2015).

References

Bailey, P., Chang, D. K., Nones, K., Johns, A. L., Patch, A. M., Gingras, M. C., Miller, D. K., Christ, A. N., Bruxner, T. J., Quinn, M. C., *et al.* (2016). Genomic analyses identify molecular subtypes of pancreatic cancer. Nature *531*, 47-52.

Bindea, G., Galon, J., and Mlecnik, B. (2013). CluePedia Cytoscape plugin: pathway insights using integrated experimental and in silico data. Bioinformatics *29*, 661-663.

Dobin, A., Davis, C. A., Schlesinger, F., Drenkow, J., Zaleski, C., Jha, S., Batut, P., Chaisson, M., and Gingeras, T. R. (2013). STAR: ultrafast universal RNA-seq aligner. Bioinformatics *29*, 15-21.

Jablonski, K. A., Amici, S. A., Webb, L. M., Ruiz-Rosado Jde, D., Popovich, P. G., Partida-Sanchez, S., and Guerau-de-Arellano, M. (2015). Novel Markers to Delineate Murine M1 and M2 Macrophages. PLoS One *10*, e0145342.

Law, C. W., Chen, Y., Shi, W., and Smyth, G. K. (2014). voom: Precision weights unlock linear model analysis tools for RNA-seq read counts. Genome Biol *15*, R29.

Liao, Y., Smyth, G. K., and Shi, W. (2014). featureCounts: an efficient general purpose program for assigning sequence reads to genomic features. Bioinformatics *30*, 923-930.

Love, M. I., Huber, W., and Anders, S. (2014). Moderated estimation of fold change and dispersion for RNA-seq data with DESeq2. Genome Biol *15*, 550.

Ritchie, M. E., Phipson, B., Wu, D., Hu, Y., Law, C. W., Shi, W., and Smyth, G. K. (2015). limma powers differential expression analyses for RNA-sequencing and microarray studies. Nucleic Acids Research *43*, e47.
